# OPENPichia: licence-free *Komagataella phaffii* chassis strains and toolkit for protein expression

**DOI:** 10.1038/s41564-023-01574-w

**Published:** 2024-03-04

**Authors:** Katrien Claes, Dries Van Herpe, Robin Vanluchene, Charlotte Roels, Berre Van Moer, Elise Wyseure, Kristof Vandewalle, Hannah Eeckhaut, Semiramis Yilmaz, Sandrine Vanmarcke, Erhan Çıtak, Daria Fijalkowska, Hendrik Grootaert, Chiara Lonigro, Leander Meuris, Gitte Michielsen, Justine Naessens, Loes van Schie, Riet De Rycke, Michiel De Bruyne, Peter Borghgraef, Nico Callewaert

**Affiliations:** 1 Center for Medical Biotechnology, VIB, Ghent, Belgium; 2 Department of Biochemistry and Microbiology, Ghent University, Ghent, Belgium; 3 Inbiose NV, Ghent, Belgium; 4 Department of Biomedical Molecular Biology, Ghent University, Ghent, Belgium; 5 BioImaging Core, VIB, Ghent, Belgium

**Keywords:** Microbiology, Molecular biology, Expression systems

## Abstract

The industrial yeast *Komagataella phaffii* (formerly named *Pichia pastoris*) is commonly used to synthesize recombinant proteins, many of which are used as human therapeutics or in food. However, the basic strain, named NRRL Y-11430, from which all commercial hosts are derived, is not available without restrictions on its use. Comparative genome sequencing leaves little doubt that NRRL Y-11430 is derived from a *K. phaffii* type strain deposited in the UC Davis Phaff Yeast Strain Collection in 1954. We analysed four equivalent type strains in several culture collections and identified the NCYC 2543 strain, from which we started to develop an open-access *Pichia* chassis strain that anyone can use to produce recombinant proteins to industry standards. NRRL Y-11430 is readily transformable, which we found to be due to a *HOC1* open-reading-frame truncation that alters cell-wall mannan. We introduced the *HOC1* open-reading-frame truncation into NCYC 2543, which increased the transformability and improved secretion of some but not all of our tested proteins. We provide our genome-sequenced type strain, the *hoc1*^tr^ derivative that we named OPENPichia as well as a synthetic, modular expression vector toolkit under liberal end-user distribution licences as an unencumbered OPENPichia resource for the microbial biotechnology community.

## Abstract (web summary)

Metabolic engineers and synthetic biologists can produce recombinant proteins at scale without royalty payments, or strain distribution constraints, using OPENPichia.

## Main

Recombinant proteins are predominantly produced by just a few different host cells. *Escherichia coli* is the main prokaryotic host used for the production of simple stable proteins that have few or no disulphide bonds. Human HEK293 cells or hamster CHO cells are used to produce more complex eukaryotic proteins that require, among other things, the formation and isomerization of disulphide bonds^1–3^ and complex-type *N*-glycosylation. Taking up the intermediate position, the methylotrophic yeast *Pichia pastoris* (reclassified as *Komagataella phaffii*) combines the easy cultivation, fast growth and highly scalable robust bioreactor processes of a microbial host with the capabilities of a eukaryotic secretory system.

**Fig. 1:**
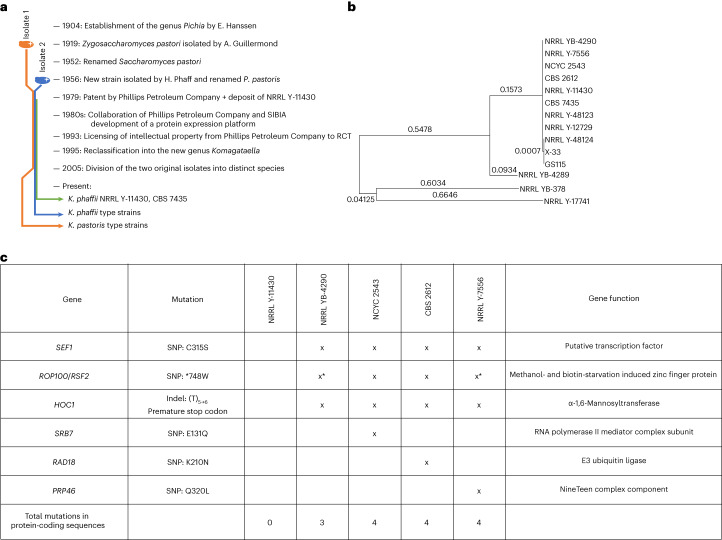
Strain overview. **a**, Schematic time line of the history of the *Komagataella* species as well as the available type-strain and patent-strain deposits. SIBIA, Salk Institute Biotechnology/Industrial Associates. **b**, Phylogenetic tree of the *K. phaffii* strains with node lengths shown. The tree was constructed using a maximum likelihood method and the Hasegawa–Kishino–Yano model. Node lengths of <0.0001 were neglected. **c**, Summary of the coding mutations in the Phillips Petroleum Company strain and type strains compared with the CBS 7435 reference genome. The specific mutations are described in the second column, with the CBS 7435 amino acid as the reference amino acid (although the original genetic makeup is at present in the type strains and it is the CBS 7435/NRRL Y-11430 that mutated). The mutations indicated with asterisks were also reported by Brady and colleagues^14^. All mutations found in these strains are concentrated in six locations. The mutations in *SEF1*, *ROP100*/*RSF2* and *HOC1* are shared by all type strains. The other mutations (in *SRB7*, *RAD18* and *PRP46*) are present in one of the type strains (NCYC 2543, CBS 2612 and NRRL Y-7556, respectively).

In 1954 H. Phaff deposited a methylotrophic yeast strain that he isolated from a black oak tree (*Quercus kelloggii*) in the Yosemite region^4^. This isolate was stored in the culture collection of the University of California at Davis and named UCD-FST K-239, with formally equivalent type-strain deposits in other culture collections named NRRL YB-4290, NRRL Y-7556, CBS 2612, NCYC 2543 and MUCL 46514. In the 1950s UCD-FST K-239 could not be distinguished from other methylotrophic yeast strains isolated in 1919 by A. Guilliermond, and Phaff categorized all isolates together as a new species named *P. pastoris* (the genus *Pichia* was established half a century before, in 1904, by E. C. Hanssen^5^; Fig. 1a). *P. pastoris* was reclassified into the genus *Komagataella* in 1995. The two distinctly evolved isolates from Phaff and Guilliermond were later (2005) divided into two separate species and renamed *K. phaffii* and *Komagataella pastoris* by C. Kurtzman^6^ based on sequencing of 26S ribosomal DNA. Consequently, the Phaff strain (UCD-FST K-239, NRRL YB-4290, NRRL Y-7556, CBS 2612, NCYC 2543 and MUCL 46514) is considered the type strain of the species *K. phaffii*, whereas the Guilliermond strain (CBS 704 and NRRL Y-1603) is the type strain of *K. pastoris*.

In the 1970s *Komagataella* yeast species, which can utilize methanol as a sole carbon source^7–9^, attracted the interest of the Phillips Petroleum Company. They had a vast supply of methane gas, which is produced during oil refinement and can be readily oxidized to methanol. The Phillips Petroleum Company isolated a *P. pastoris* strain that fermented methanol to form a single-cell protein source for animal feed and patented this application in 1980 (with a priority date of 12 April 1979)^10^. Patenting included a requirement for strain deposition, with the patented strain being named NRRL Y-11430 (known as CBS 7435 in a different culture collection). The Phillips Petroleum Company contracted the Salk Institute Biotechnology/Industrial Associates in the 1980s to develop NRRL Y-11430 for recombinant protein production. NRRL Y-11430-derived strains were generated by nitrosoguanidine mutagenesis, resulting in (among other things) the GS115 strain, which is a *HIS4* auxotrophic mutant^11^, and the X-33 strain, which is a *HIS4*-complemented GS115 produced by Invitrogen^11–13^. Phillips Petroleum sold the patent rights for their *Pichia* system to Research Corporation Technologies (RCT; https://pichia.com/) in 1993. Surprisingly, NRRL Y-11430 (Agricultural Research Service Culture Collection, ARS-NRRL) is not distributed anymore by NRRL and the same holds for the equivalent CBS 7435 deposit (Westerdijk Fungal Biodiversity Institute, CBS). To our knowledge, the NRRL Y-11430 parental industrial strain can only be obtained at the American Type Culture Collection (ATCC 76273) under a restrictive material transfer agreement (MTA) precluding third-party distribution and use for product manufacturing. Derivative industrial strains (GS115 and X-33) have similar restrictions when licensed from the providing companies. Royalty payments are imposed on products manufactured in them.

Hence, despite the expiry of the associated patent more than 20 yr ago, socio-economic utilization of the parental NRRL Y-11430 strain and its derivatives in this way remains monopolized through a commercial licensing scheme. The lack of freedom to distribute the result of synthetic biology efforts in academia and industry alike to enhance the capabilities of *Pichia* strains greatly impedes progress with this cornerstone system of recombinant protein biotechnology. An equivalent open-access alternative is hence long overdue.

Researchers in academia and industry ideally need to use the same parental *Pichia* strain lineage that has already been commercialized because regulatory agencies are familiar with this strain. To achieve this goal, we and others have recently turned to genome sequencing of the *K. phaffii* type strains that are present in culture collections throughout the world to try and identify the original isolate from nature that the Phillips Petroleum Company researchers used in their derivation of NRRL Y-11430, as the basis from which an open-access system could be built^14^. Here we resequenced genomes of four type strains and selected the NCYC 2543 deposit for development as a chassis strain. This equivalent deposit of the Phaff UCD-FST K-239 type strain is genomically near-identical to NRRL Y-11430, consistent with it being the parent type strain, and the NCYC collection provides liberal distribution and commercial use licences. We exhaustively compare the biological features of NCYC 2543 to the NRRL Y-11430 industrial strain, and engineer an optimized derived ‘OPENPichia’ strain that is equally performant as the industrial strain.

We present OPENPichia together with a modular protein expression vector toolkit completely built from synthetic DNA, free of third-party MTAs, that is compatible with toolkits from other *Pichia* developer laboratories^15^ as a resource for the global microbial metabolic engineering and synthetic biology communities.

## Results

### Genome resequencing of *K. phaffii* strains

We resequenced (average of 180× genome coverage) NRRL YB-4290, NRRL Y-7556, CBS 2612, NCYC 2543 and the NRRL Y-11430 industrial strain. The reads were mapped against the reference genome (CBS 7435), which includes the mitochondrial genome and two *K. phaffii* linear killer-like plasmids^12^ (Supplementary Tables 1–3).

The proportion of reads originating from the two killer-like plasmids varied between 0% and 9% (Supplementary Table 3). *K. phaffii* killer-like plasmids are linear autonomously replicating DNA fragments with a length of 9.5 and 13.1 kilobases (kb)^12^ that place a biosynthetic load on cells and also encode exotoxins that can kill yeast cells^12,16^, which might conceivably reduce culture viability. Killer-like plasmids were absent from CBS 2612 and NCYC 2543 but present in NRRL YB-4290, Y-7556 and Y-11430 (Supplementary Table 4). The NRRL YB-4290 and CBS 2612 strains were deposited by Phaff, whereas the NRRL Y-7556 strain was a re-deposit of CBS 2612 by D. Yarrow (CBS; Fig. 1a). Given that NRRL Y-7556 has killer-like plasmids but CBS 2612 does not, it is clear that killer-like plasmids can be lost frequently in vitro simply by propagation and single-clone purification.

A phylogenetic tree of resequenced strains (this study) and previously published *K. phaffii* genomes^11,14,17^ showed that *K. phaffii* type strains are clustered with NRRL Y-11430, CBS 7435 and close relatives (Fig. 1b). Our data support the previously published hypothesis^14,18^ that all deposited *K. phaffii* strains are derived from the Phaff isolate^17^.

To identify an equivalent type strain to NRRL Y-11430, we identified single nucleotide polymorphisms (SNPs) and short insertion–deletions (indels) in our resequenced strains (Supplementary Table 4). We detected approximately 20 intergenic/intronic/silent exonic differences between NRRL Y-11430 and CBS 7435. Note that the type-strain deposits of the different culture collections (NRRL YB-4290, NCYC 2543, CBS 2612 and NRRL Y-7556) also differ from one another, each at one other coding sequence-altering genomic position and a few non-coding ones, probably reflecting drift due to the background mutational rate during strain propagation (Fig. 1c).

We focused on protein-coding alterations that consistently distinguish the industrial strain NRRL Y-11430 from these equivalent type-strain deposits. Three coding sequence-altering mutations (in *SEF1*, *RSF2* and *HOC1*) were shared by all type strains but were absent from the industrial strain NRRL Y-11430. We re-analysed raw sequencing reads from a previous characterization of NRRL YB-4290 and NRRL Y-7556, and confirmed the presence of *SEF1*, *RSF2* and *HOC1* mutations^14^. As all three mutations are shared by the type strains, we conclude that they represent the original *K. phaffii* isolate and that NRRL Y-11430 is mutated at these loci.

### *SEF1*, *RSF2* and *HOC1* genotypes in NRRL Y-11430 and CBS 7435

*SEF1* encodes a putative transcription factor (UniProt ID F2QV09). The SNP causes a S315C mutation in NRRL Y-11430. *RSF2* encodes a transcription factor that is involved in methanol- and biotin-starvation (UniProt ID F2QW29). The SNP introduces a stop codon (W748*) in NRRL Y-11430, resulting in a carboxy (C)-terminal deletion of 183 amino acids. Full-length Rsf2p is similar to a *Saccharomyces cerevisiae* homologue^19^, providing support for the idea that this was the original genomic state, as previously reported^14^. *HOC1* (*OCH1* homologue) encodes an α-1,6-mannosyltransferase (UniProt ID F2QVW2) involved in the synthesis of cell-wall mannan and is part of the mannan polymerase II complex^20^. The industrial strain NRRL Y-11430 has a single base pair (bp) deletion in a poly-A stretch (at position 755 of the 1,191 bp coding sequence). This is predicted to result in a C-terminally truncated protein (274 versus 398 amino acids), with the last 22 codons after the frameshift and before the first-occurring stop codon coding for an altered C-terminal peptide. We confirmed the indel in the homopolymer using Sanger sequencing (Supplementary Data 1). In parallel, the same mutation was identified in K. Wolfe’s laboratory (UC Dublin) as a quantitative trait locus mutation that yielded between two- and three-fold higher secretion of a β-glucosidase that was used as a secretion reporter protein^18^.

### Growth rate and protein production differences between strains

**Fig. 2:**
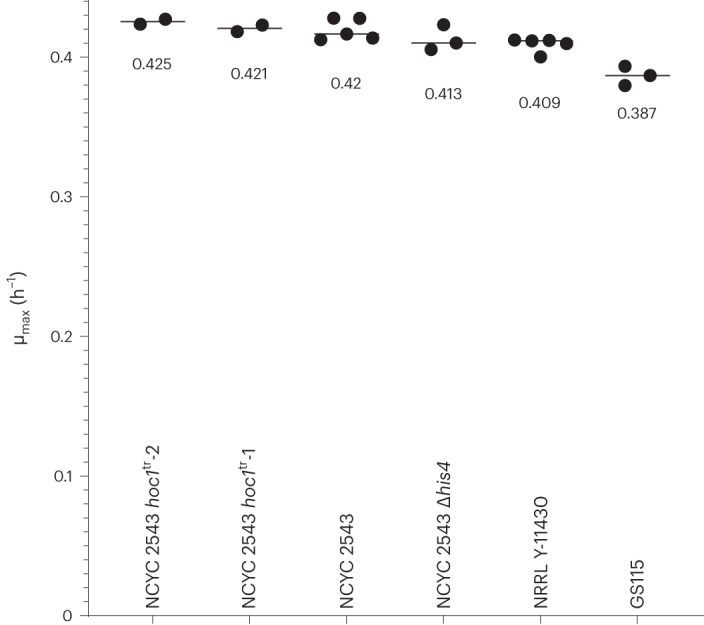
Comparison of the maximal growth rate of NRRL Y-11430, NCYC 2543, the two NCYC 2543 *hoc1*^tr^ mutants, GS115 and NCYC 2543 Δ*his4*. The data points represent technical replicates (2 ≤ *n* ≤ 5) and the median of each group is represented with a horizontal line. Source data

Next, we compared characteristics that are important for the use of *K. phaffii* in recombinant protein production and focused on NCYC 2543 given the availability of open-access distribution options by the NCYC culture collection (https://www.ncyc.co.uk/licences). We compared the growth rates of NRRL Y-11430, GS115, NCYC 2543 and a NCYC 2543 *HIS4*-knockout mutant (NCYC 2543 Δ*his4*; Fig. 2). GS115 grew significantly slower, as reported earlier^14^. Given that the NCYC 2543 Δ*his4* strain did not grow slower than its wild-type counterpart, the slower growth of GS115 is not, or at least not only, due to histidine auxotrophy.

**Extended Data Fig. 1:**
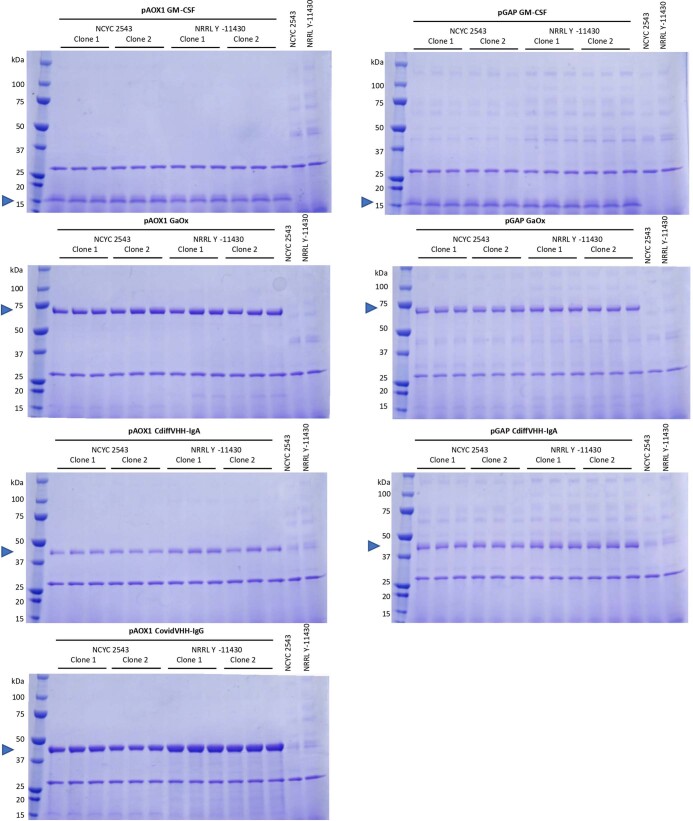
Expression comparison between NCYC 2543 and NRRL Y-11430. The proteins were expressed using the *GAP* or *AOX1* promoter (three biological replicates per strain, promoter and protein). As controls, both wild type strains were grown and analysed as well. Supernatant samples were treated with EndoH to remove N-glycans and samples were analysed on SDS–PAGE. EndoH is also visible on the gels at around 30 kDa. The arrows indicate the expected location of the expressed protein band, based on the theoretical size. Source data

**Extended Data Fig. 2:**
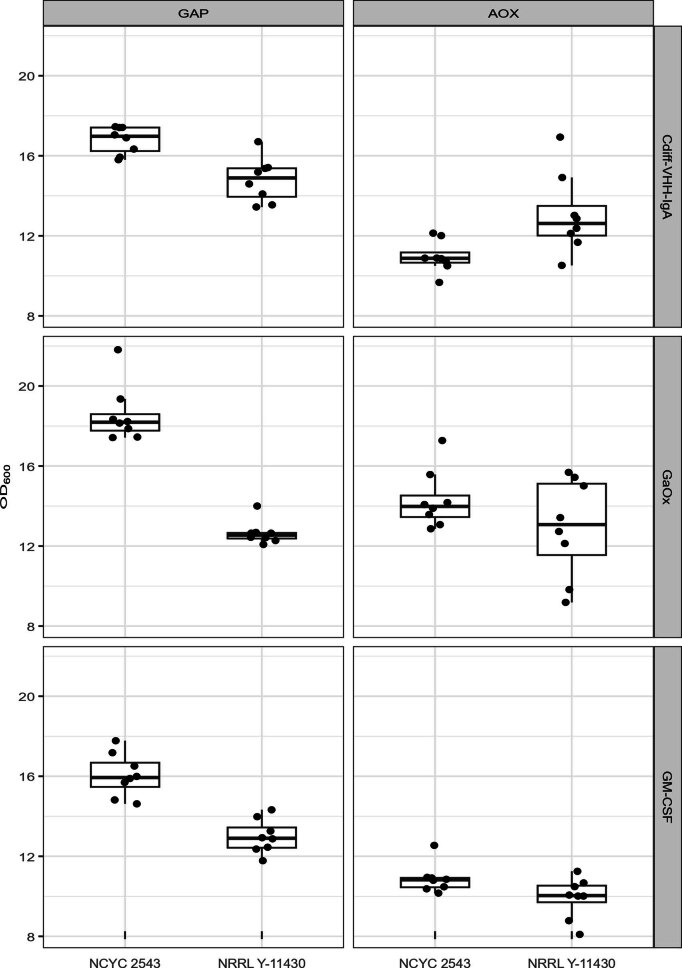
Summary of the end-ODs of the pGAP- and pAOX1-based cultivations at harvest. For both strains, NCYC 2543 and NRRL Y-11430, the end-ODs for the three model protein cultivations are depicted. Data points for the biological replicates (n = 2) were determined from four technical replicates. Box plots elements: centre line, median; bottom and top lines, lower and upper quartiles; whiskers, maximally 1.5× the interquartile range, or less when no data points are outside this distance. Source data

We expressed a selection of proteins (Supplementary Table 5) in NRRL Y-11430 and NCYC 2543 to evaluate how well NCYC 2543 expressed recombinant proteins. We chose four proteins that exemplify the different protein types produced by biotechnology companies: a cytokine (GM-CSF), a redox enzyme (GaOx), a VHH-hFcα fusion (Cdiff-VHH-IgA) and a VHH-hFcγ fusion (CovidVHH-IgG). We tested two promoters—that is, glyceraldehyde 3-phosphate dehydrogenase promoter (PGAP; constitutive) and alcohol oxidase I promoter (PAOX1; methanol-inducible). Protein expression in *K. phaffii* is prone to clonal variations that can interfere with the comparison of expression capabilities between strains, mostly due to the integration site and the copy number of the construct^21^. To overcome this problem, a single-copy of the cloned gene was targeted to specific promoter regions in the genome. We confirmed copy number and integration sites by quantitative and integration-site-specific PCR, and two independent clones that expressed each of the four proteins were cultured in triplicate. Similar amounts of proteins were produced by both the PGAP and PAOX1 constructs (Extended Data Fig. 1), with the exception of the VHH-hFcγ fusion, where NRRL Y-11430 outperformed NCYC 2543. However, NCYC 2543 harbouring PGAP constructs grew to higher densities than NRRL Y-11430 harbouring PGAP constructs (Extended Data Fig. 2), whereas this was not the case for PAOX. In addition, NRRL Y-11430 harbouring PGAP constructs (in limiting glucose) produced more host cell proteins than NCYC 2543 harbouring PGAP (Extended Data Fig. 1). We hypothesize that a low level of cell lysis or protein leakage occurs in NRRL Y-11430 cultured on glucose.

### *HOC1* truncation restores NCYC 2543 transformation efficiency

**Fig. 3:**
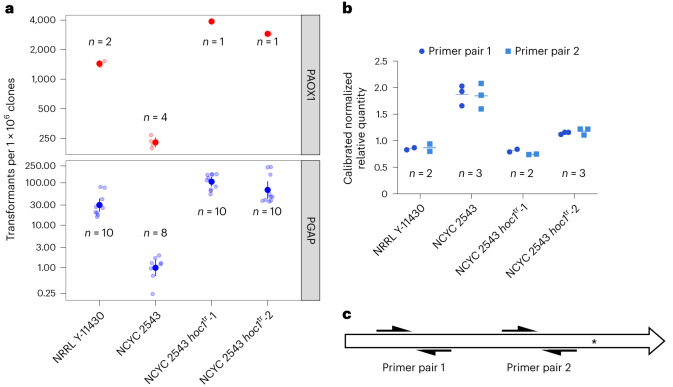
Effect of the *HOC1* truncations on plasmid transformation efficiency and *HOC1* mRNA abundance in the resulting strains. **a**, Transformation efficiency of a PAOX1- (top) and a PGAP-based plasmid (bottom) in the two wild-type and two *HOC1*-engineered strains. The analysis was performed as described in Methods. Technical repeats (1 ≤ *n* ≤ 10) are shown as semi-transparent data points; the solid data points and error bars are the group means and the 95% confidence intervals (where available), respectively, as estimated by the linear model. **b**, Levels of *HOC1* mRNA, as determined using RT–qPCR, in the different strains. Individual biological repeats (*n* = 2 or 3) and their mean are shown as points and bars. Each biological replicate was determined from three technical replicates. **c**, Schematic representation of the primers used in the RT–qPCR experiment: primer pair 1 binds near the start codon of *HOC1*, whereas primer pair 2 binds close to the premature stop codon. The asterisk indicates the position of the premature stop codon in NRRL Y-11430 and the *HOC1*-truncated mutants of NCYC 2543. The stop codon is absent in the NCYC 2543 strain. Source data

**Extended Data Fig. 3:**
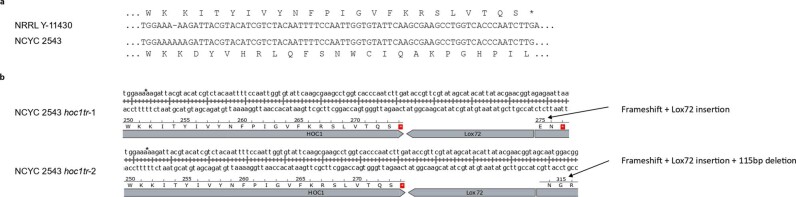
Overview of the *HOC1* genome engineering strategy. **a**. Alignment of a part of the *HOC1* gene as present in NRRL Y-11430 vs. NCYC 2543, showing the frameshift resulting in a premature stop codon in the NRRL Y-11430. **b**. Resulting genomic *HOC1* sequence upon split-marker-based gene editing. Two strategies were followed where either the single base pair deletion (indicated with *) resulting in the Hoc1p truncation and a Lox72 scar is introduced downstream of the stop codon; or where an additional 115 bp deletion downstream of the resulting stop codon and Lox72 scar is introduced.

The transformation efficiency of NCYC 2543 was only 16% (95% confidence interval, 13–19%) and 3% (95% confidence interval, 2–7%) compared with NRRL Y-11430 for PAOX1 and PGAP expression constructs (Fig. 3a), which is consistent with the low transformation efficiency of the type strains that was reported previously^14,18^. As the *S. cerevisiae* Hoc1p orthologue is an α-1,6-mannosyltransferase that functions to produce the outermost layer of the ascomycete cell wall, we hypothesized that a reduced diffusional/charge barrier, due to reduced mannan/mannosylphosphate density, might explain the superior transformability of NRRL Y-11430. Using the split-marker method, we introduced a single base pair deletion in *HOC1* of NCYC 2543 (Extended Data Fig. 3a) to produce NCYC 2543 *hoc1*^tr^-1 and a larger deletion to remove 115 bp downstream of the novel stop codon to produce NCYC 2543 *hoc1*^tr^-2 (Extended Data Fig. 3b). We used quantitative PCR with reverse transcription (RT–qPCR) to measure the production of *HOC1* messenger RNA and found that *HOC1* transcription was downregulated in the strains with a premature stop codon (Fig. 3b). The NCYC 2543 *hoc1*^tr^-1 strain, in which the premature stop codon is separated from the canonical stop codon by 405 nucleotides (nt), produced the lowest level of transcripts. The NRRL Y-11430 and NCYC 2543 *hoc1*^tr^-2 strains have 371 and 290 nt, respectively, between the premature and the canonical stop codon, which correlates with transcript abundance. In conclusion, *HOC1*-truncated strains lack part of the C-terminal catalytic domain and probably also contain less Hoc1p in the mannan polymerase complex. We compared the transformation efficiency of both wild-type strains and *hoc1*^tr^ mutants and found that the *HOC1* truncation strongly increased the transformation efficiency of the type strain and even surpassed the transformation frequency by 1.5–3-fold compared with NRRL Y-11430 (Fig. 3a).

### Cell walls of NRRL Y-11430, NCYC 2543 and NCYC 2543 *hoc1*^tr^

**Fig. 4:**
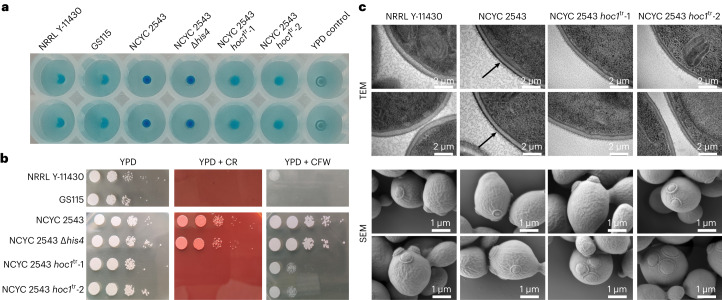
Characterization of the cell walls of NRRL Y-11430, NCYC 2543 and the two NCYC 2543 *hoc1*^tr^ mutants. **a**, Alcian blue staining of the strains to determine the density of negative charges at the yeast cell wall. Alcian blue is a cationic dye that binds negative charges at the cell wall. The more intense the blue staining of the cells, the more negative charge—that is, mannosylphosphate moieties—are present on the glycan trees of the cell-wall mannoproteins. Duplicate wells are shown for each strain (vertical). **b**, Sensitivity of the strains to Congo red (CR) and Calcofluor white (CFW), compared with growth on YPD agar, as an indicator of the cell-wall integrity. The plates were incubated at 30 °C for 3 days. **c**, Transmission (TEM) and scanning electron microscopy (SEM) images of the four strains. The increased electron density of the outermost layer—that is, the cell wall—is indicated with an arrow in the TEM images of the NCYC 2543 strain. Only two individual images per strain are shown.

**Extended Data Fig. 4:**
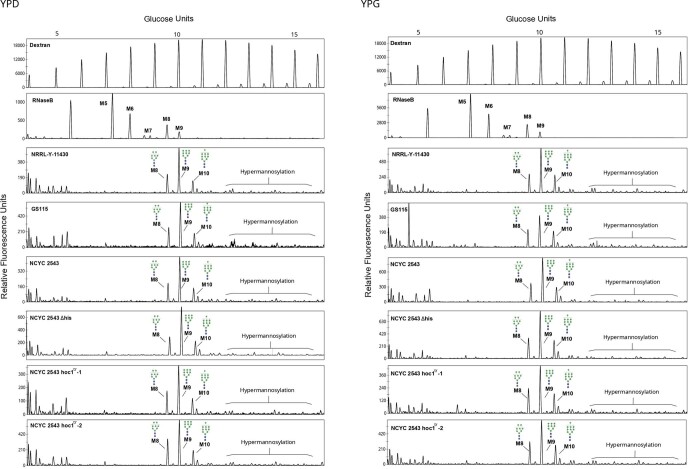
CGE-LIF profiles of the cell wall mannoproteins of the different strains grown on YPD or YPG. N-glycan profiles of the cell wall mannoproteins when the strains were grown on YPD (left panels) vs. on YPG (right panels). The predominant peaks are Man_8_GlcNAc_2_ (M8), Man_9_GlcNAc_2_ (M9), and Man_10_GlcNAc_2_ (M10).

We characterized cell-wall mannoprotein *N*-glycans using capillary electrophoresis^22^ after growth on glucose or glycerol (Extended Data Fig. 4). All four strains had very similar profiles, indicating that the pathway of synthesis of the mannan core was intact. This capillary electrophoresis method is unsuited to detailed profiling of higher-polymerized mannan *N*-glycans. Most mannosylphosphates are added to the mannan side branches of these long chains, which makes them bind to cationic dyes such as Alcian blue. Hence, we compared the Alcian blue staining intensity of NCYC 2543, NRRL Y-11430 and the two type-strain *hoc1*^tr^ mutants (Fig. 4a). Reduced Alcian blue staining of the latter was consistent with that of published *S. cerevisiae hoc1* strains^23,24^.

The resistance of strains to Congo red and Calcofluor white was analysed to assess their cell-wall integrity (Fig. 4b)^14,18^. The type strain was more resistant than NRRL Y-11430 to both dyes but this difference was absent in the *HOC1*-truncated mutants, which shows that Hoc1p contributes to cell-wall integrity. Transmission electron microscopy using a freeze substitution technique (which draws OsO_4_ membrane-staining contrast reagent and fixatives through the cell wall during the dehydration of cells) revealed increased electron scattering by the outermost cell-wall layer of the wild-type NCYC 2543 strain compared with the *HOC1*-truncated strains (Fig. 4c). This is probably caused by OsO_4_ accumulation in the mannan layer of the cell wall during freeze substitution. Scanning electron microscopy analyses indicated that all four strains were structurally similar (Fig. 4c), indicating the absence of gross malformations. We conclude that the *hoc1*^tr^ mutation results in a mild deficiency in cell-wall integrity, which increases transformability and in some cases increases the production or secretion of recombinant proteins^18^.

### Protein production by NCYC 2543 *hoc1*^tr^

**Fig. 5:**
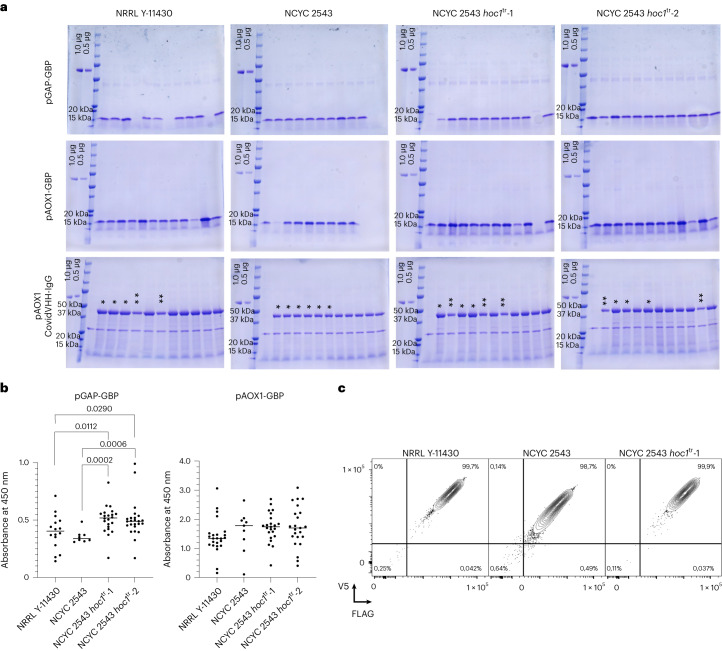
Overview of the strain performance of NRRL Y-11430, NCYC 2543 and the two NCYC 2543 *hoc1*^tr^ mutants. **a**, SDS–PAGE analysis of the first 12 randomly picked transformants of pGAP-GBP, pAOX1-GBP and pAOX1-CovidVHH-IgG. Equal volumes of supernatant were loaded. For pAOX1-CovidVHH-IgG expression, the copy number of some clones were determined: each asterisk represents one copy integrated into the genome. **b**, ELISA results of 24 clones (individual points represent biological replicates) and the median (lines) of PGAP- (left) and PAOX1-based (right) GBP expression in the different strains (no selection for single copies was done; wells were excluded when no expression was observed on SDS–PAGE gels, assuming these clones contain no expression cassette, or due to a technical issue during the ELISA procedure). The absorbance units were background corrected. All strains were compared using a Kruskal–Wallis omnibus test, followed by pairwise comparison corrected using Dunn’s multiple comparison procedure. Significance scores are provided in the graph; non-significant differences are not shown. **c**, Example of a surface display expression comparison (data of additional clone and replicates in Extended Data Fig. 5). Human lysozyme was fused to the C-terminal part of Sag1 (which contains a glycosylphosphatidylinositol anchor) as well as an N-terminal FLAG tag and a C-terminal V5 tag for detection in flow cytometry. The resulting fusion protein was expressed using the *AOX1* promoter. Cells were plotted by a 5% quantile contour plot, with outliers presented as dots. Quadrant gates were set using unstained and single-stained controls, and the percentage of cells in each quadrant is indicated. Source data

The growth rates and protein production capacities of the NCYC 2543 *hoc1*^tr^ strains were compared with NRRL Y-11430 and NCYC 2543. No significant difference in growth rate was observed (Fig. 2). We tested the PGAP- and PAOX1-based production of GBP and CovidVHH-IgG in the supernatant using SDS–polyacrylamide gel electrophoresis (SDS–PAGE) and enzyme-linked immunosorbent assay (ELISA; GBP only). We screened 24 clones of each strain with the exception of the type strain, where only 11 and 9 transformants were obtained for PAOX1- and PGAP-constructs, respectively (Fig. 5a,b).

The NCYC 2543 *hoc1*^tr^ strains outperformed both NRRL Y-11430 and NCYC 2543 in the production of GBP protein from the PGAP promoter, although the differences were small and clonal distributions overlapped. These results are consistent with published data^18^. No statistically significant differences in PAOX1-GBP protein production were observed between strains. For PAOX1-CovidVHH-IgG production and secretion, NCYC 2543 produced reduced yields compared with the *hoc1*^tr^ strains (Fig. 5a). However, we observed two classes of production levels, raising the question of copy number effect. We determined that single-copy insertions resulted in higher CovidVHH-IgG production than double-copy insertions (Fig. 5a; each asterisk represents one copy in the tested clone). Given that this was observed for all four tested strains, it is likely to be an effect of this specific protein rather than the host.

**Extended Data Fig. 5:**
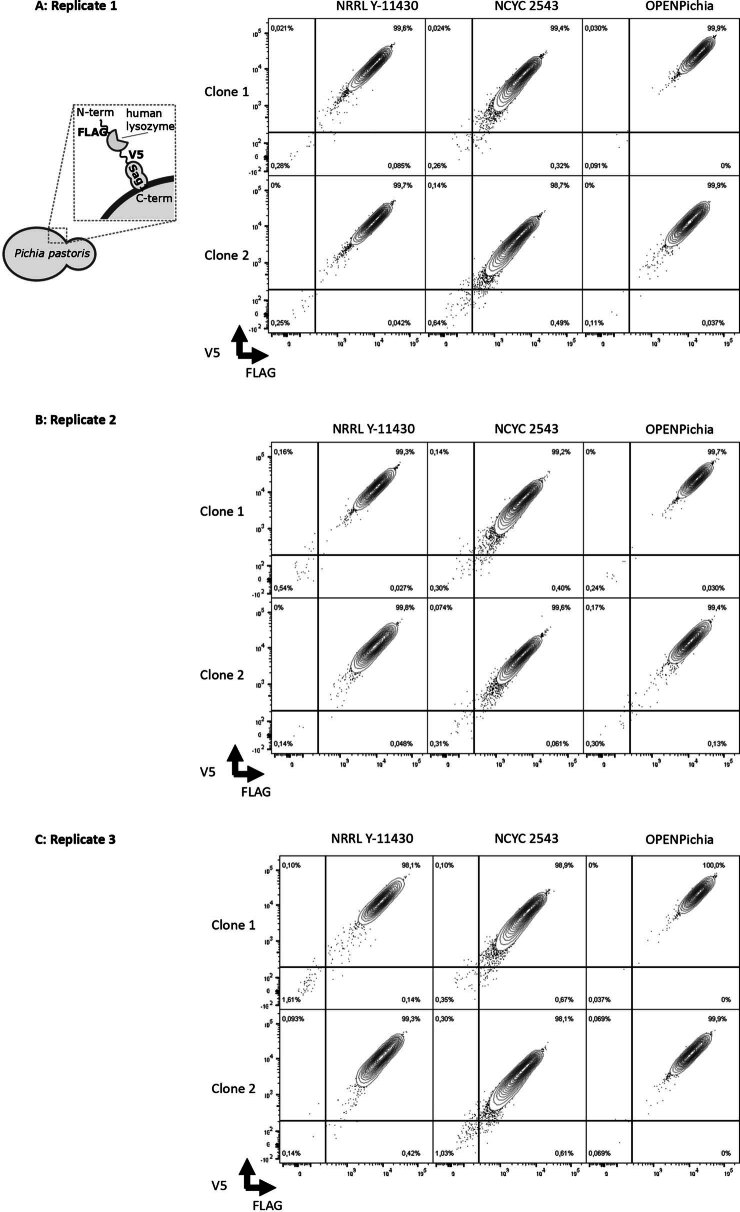
Surface display phenotype of *Pichia pastoris* strains NRRL Y-11430, NCYC 2543 and OPENPichia. Human lysozyme was fused to the C-terminal part of Sag1p (which contains a GPI anchor) and with an N-terminal FLAG tag and a C-terminal V5 tag for detection in flow cytometry. The resulting fusion protein was expressed using the *AOX1* promoter. Copy number was determined and two clones with a copy number of 1 were selected for each strain. Cells were plotted by a 5% quantile contour plot with outliers presented as dots. Quadrant gates were set using non-stained and single-stained controls. The number of technical replicates is 3. The gating strategy is shown in Extended Data Fig. 6.

**Extended Data Fig. 6:**
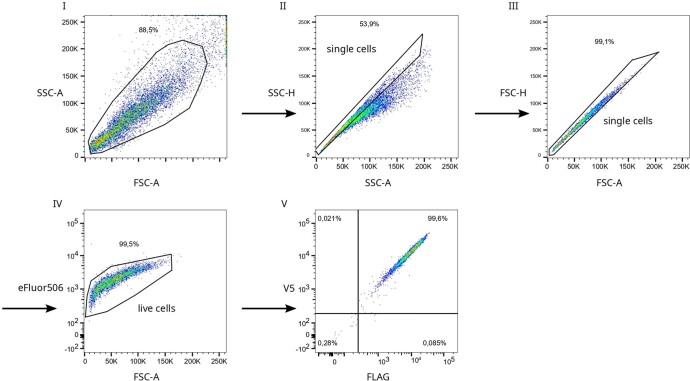
Gating strategy of the yeast surface display experiment. At least 10,000 events are captured on a FACSMelody instrument and data is analysed with the FlowJo software. First, debris is gated out, then single cells are gated using both the side scatter (SSC) and the forward scatter (FSC), and finally living cells are gated by using the Live/Death stain eFluor506. The resulting living single *Pichia* cells are analysed based on FLAG and V5 signal. The gates of IV and V were determined based on non-stained and single-stained controls.

Next, the expression and surface display of an amino (N)-terminal FLAG-tagged and C-terminal V5-tagged human lysozyme were evaluated. By detecting the tags on both sides of the protein (Fig. 5c and Extended Data Figs. 5, 6), we observed a similar intensity of detection in NRRL Y-11430 and NCYC 2453 *hoc1*^tr^-1, and reduced detection in NCYC 2543, showing that the truncated *hoc1* allele is beneficial for protein surface display and/or the ease of detection of the displayed protein using antibody detection reagents.

**Extended Data Fig. 7:**
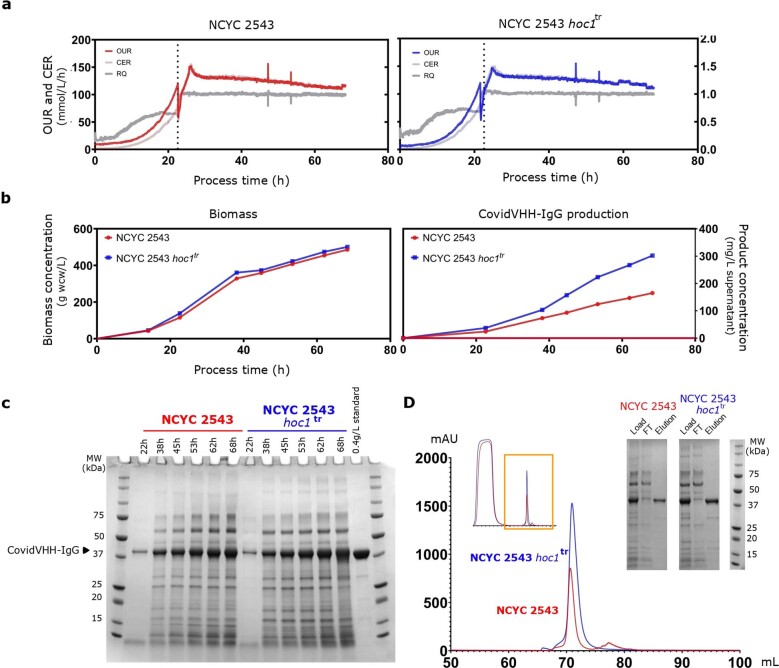
Comparative analysis of NCYC 2543 and NCYC 2543 *hoc1*^tr^ strains expressing CovidVHH-IgG in a 3 l fermenter. Both strains, expressing the protein with a *GAP* promoter were compared in a fed-batch production in a 3 l fermenter. Note that NCYC 2543 had a single-copy insertion, while NCYC 2543 *hoc1*^tr^ had a double-copy insertion of the CovidVHH-IgG expression cassette. They have comparable growth kinetics (batch phase length, oxygen demand and biomass formation), but NCYC 2543 *hoc1*^tr^ produced more protein. **a**. Profile of oxygen uptake rate (OUR), carbon exchange rate (CER) and resipiration quotient (RQ). The end of the batch phase and start of the feed phase is indicated with a vertical dotted line **b**. Biomass and protein production kinetics. Biomass data points are the mean of triplicate technical repeat measurements of the same fermentation run. Protein concentrations are single measurements as determined from the Äkta A260nm measurements. **c**. SDS–PAGE analysis of time-course samples collected from the fermentation runs. Samples are labelled with hours elapsed since the start of the fermentation process. Equivalent volumes of supernatant were loaded into each well for analysis. **d**. Comparison of ProteinA elution peaks from the ÄKTA chromatograms of harvest samples from both fermentation runs (a single replicate per strain is shown). To verify the successful capture of the entire product from the supernatants, purification fractions (load, flow through (FT), and elution) were analysed with SDS–PAGE. Source data

To evaluate whether *HOC1*-truncated NCYC 2543 can be cultured to a high cell density in bioreactors, we compared NCYC 2543 *hoc1*^tr^-1 and NCYC 2543 in a fermentation experiment at a 3 l scale. We chose CovidVHH-IgG as the target protein, under control of PGAP. As a result of the higher transformation efficiency, the *HOC1*-truncated strain had a double-copy insertion, whereas the type-strain NCYC 2543 only had a single-copy insertion. In this experiment both strains had comparable growth (Extended Data Fig. 7). The batch phase length, oxygen demand, growth rate and final cell density were very similar. NCYC 2543 *hoc1*^tr^-1 produced almost double the amount of protein produced by the parental strain. We concluded that truncation of the *HOC1* gene does not negatively influence the performance of *K. phaffii* in a bioreactor.

In conclusion, the *HOC1* truncation did not have a negative effect on protein production in any of our experiments and sometimes yielded better production. As reported recently by Brady et al.^14^, issues with transformability, which makes it laborious to generate multicopy integration clones, was the key reason they opted for continued use of NRRL Y-11430-based strains.

We have solved this problem, and hereby rename NCYC 2543 *hoc1*^tr^-1 as OPENPichia.

### OPENPichia modular protein expression vector toolkit

Commercial *K. phaffii* expression kits containing NRRL Y-11430-derived strains as well as expression vectors are commonplace because they are convenient and work well. The conditions of sale of these kits are legally restrictive and forbid further distribution and reutilization of both the strains and the vectors included in them, including use in commercial production. Importantly, commercial applications require licensing from the kit provider, which can take time and incur costs. To also overcome issues with these proprietary DNA constructs, we used de novo synthesis combined with rapid cloning methods^25^. The development of a robust genetic toolkit with ‘freedom to operate’ is still expensive and time-consuming.

**Fig. 6:**
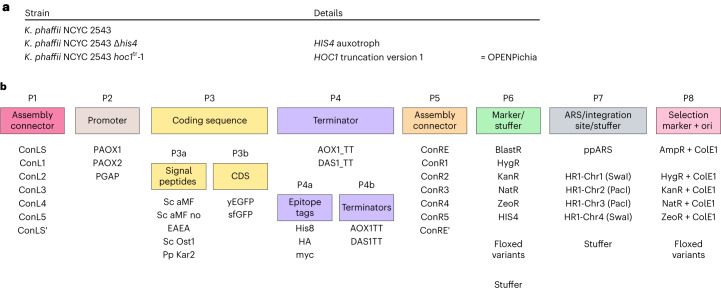
Overview of the available OPENPichia strains and the different parts of the MoClo toolbox. **a**, Overview of the strains that are available from VIB as part of the OPENPichia resource. **b**, Overview of the parts of the MoClo toolbox that is distributed from the OPENPichia resource. All of these strains and materials are new and reported for the first time in this study. For each part, the available elements are depicted. To generate multigene destination vectors or co-expressing vectors, additional parts are available. All plasmids and plasmid maps are available at the BCCM/GeneCorner Plasmid Collection.

**Extended Data Fig. 8:**
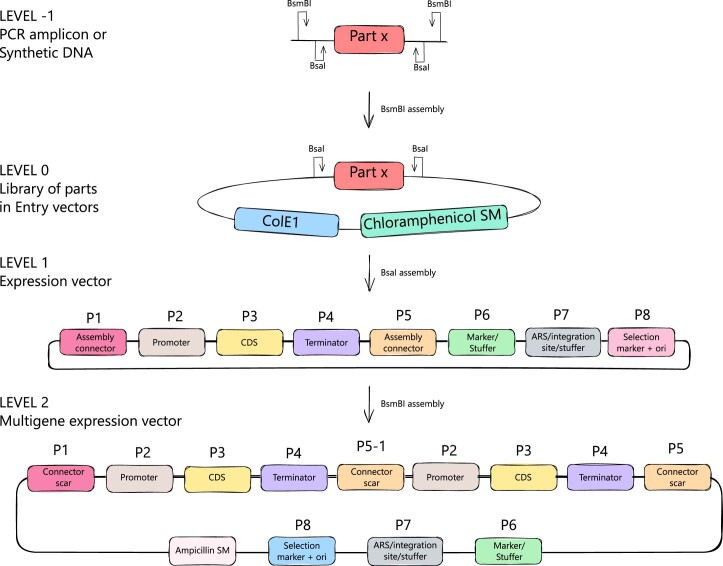
The modular cloning or MoClo principle. In Level -1, source DNA, such as PCR fragments or synthetic DNA are flanked with the proper Type IIS restriction sites and 4 nt overhangs, which are then accommodated in a Level 0 Entry vector through BsmBI digest and T4 DNA ligation. Then, selected Level 0 vectors are assembled into a Level 1 Expression vector by means of a BsaI digest and T4 DNA ligation. Finally, the system allows the assembly of multiple transcription units (promoter, CDS, terminator) from the individual Level 1 vectors, into a higher order Level 2 vector, in case the assembly connector sequences were properly selected during the assembly of the Level 1 vectors. Note that the Part 3 Coding Sequence can be split up in a Part 3a and Part 3b, to allow additional modularity. Likewise, the Part 4 Terminator can be split up in a Part 4a and a Part 4b.

We provide a genetic toolkit and cloning framework to the community (Fig. 6 and Extended Data Fig. 8)^26^. We used a modular build based on Golden Gate cloning, similar to other toolkits^15,27–35^. Golden Gate assembly is based on the use of Type IIS restriction endonucleases that cut outside their recognition sites, which allows users to flank DNA fragments of interest with customizable 4 nt overhangs, enabling directional multi-insert cloning in a single reaction. The MoClo system takes this concept a step further as it standardizes Golden Gate assembly by designating a priori all DNA elements of a desired vector, which are typically referred to as ‘parts’, to a particular ‘part type’ (for example, promoter, coding sequence and so on) and flanking each part type by unique 4 nt overhangs and Type IIS restriction sites^35^. The MoClo system is comprised of eight part types, of which Part 3 (coding sequence) and Part 4 (terminator) can be split up to allow additional modularity—for example, to incorporate N- and C-terminal fusion partners for the protein of interest. In practice, parts are derived from PCR fragments or synthetic constructs, which are first subcloned in entry vectors, also known as ‘Level 0’ vectors (Fig. 6). The vectors of interest can then be assembled into expression vectors, which are termed ‘Level 1’ vectors. By providing proper connector sequences with additional Type IIS restriction sites, the resulting Level 1 vectors can then be further assembled to obtain multigene or ‘Level 2’ vectors, which is the top level in the hierarchy of the system. In the current toolkit, all 4 nt overhangs were adopted to ensure a high degree of compatibility with existing yeast toolkits^15,28,32^ and ensure a near 100% predicted ligation fidelity^36^. As this toolkit is essentially derived from the *S. cerevisiae* MoClo system, it shares the restriction enzymes (BsmBI and BsaI), most of the 4 nt overhangs as well as the number and design of the individual part types^28^. An overview of the part types and the parts that are provided in our OPENPichia toolkit is presented in Extended Data Fig. 8. Part sequences are presented in Supplementary Data 2 and materials can be obtained from the Belgian Coordinated Collections of Microorganisms (BCCM)/GeneCorner Plasmid Collection^26^. We custom-built an MTA in collaboration with GeneCorner to enable the use of all of these plasmids, thereby making royalty-free commercial manufacturing possible.

## Discussion

*K. phaffii* (formerly known as *P. pastoris*) is an important protein production host in both academia and industry but the most common industrially developed strains are still distributed with restrictive MTAs and/or commercial licensing, despite the associated patents having expired decades ago. To facilitate academic and commercial host strain development for recombinant protein expression and enable distribution throughout the biotechnology community, we derived an OPENPichia strain and OPENPichia vector cloning kit that enables royalty-free commercial manufacture of *K. phaffii* products. The OPENPichia strains are distributed by our non-profit research organization, VIB (OPENPichia.com) in an arrangement with the NCYC culture collection. A one-time fee is charged to cover expenses as well as continued resource maintenance and development, following which any use is allowed, including royalty-free commercial product manufacture and onward distribution of further-engineered OPENPichia-derived strains. The OPENPichia vector cloning materials are openly distributed for any purpose by the BCCM (http://bccm.belspo.be/about-us/bccm-genecorner).

Our OPENPichia strain (*HOC1*-truncated *K. phaffii* type strain) is almost identical to the former patent-deposit NRRL Y-11430 strain. Only a handful of mutations were identified in comparative genome analyses, of which only four alter the protein code (SNPs and indels). OPENPichia does not harbour killer-like plasmids and its maximum growth rate is the same as that of NRRL Y-11430. With respect to protein production, small differences can occur between the *K. phaffii* type strain and NRRL Y-11430 but there is no consistently better performing strain, considering the variety of proteins tested in our study. Brady and colleagues^14^ previously reported that NRRL Y-11430 had the highest levels of protein expression compared with other *K. phaffii* strains but none of the type strains from which NRRL Y-11430 was derived were included in their study. Due to the increased cell-wall robustness and reduced transformation efficiencies of type strains, they were excluded from the protein expression experiments performed by Brady and colleagues^14^. We indeed observed that the transformation efficiency of the type strain is reduced compared with NRRL Y-11430 but we overcame this through the introduction of a frameshift mutation in *HOC1* of the type strain, which resulted in improved transformation efficiency compared with NRRL Y-11430.

Using PGAP-controlled gene expression, NRRL Y-11430 has somewhat more host cell proteins in its culture supernatant and grows to a lower cell culture density (in shake flasks) compared with the type strain. We hypothesize that both observations are related and due to slightly increased cell lysis in NRRL Y-11430, which can have an impact on the need for additional purification steps. A similar observation was made for the *HOC1*-truncated type strains, although the differences were very small.

Our study shows how to build ‘generic’, robust, validated and openly available biotechnological platforms after patents on foundational strains expire, rather like the development of more affordable ‘generic/biosimilar’ medicines. We previously reported a similar effort for the HEK293 cell lineage^37^ that is used for viral vector and vaccine manufacturing and hope that others will join us in open science endeavours to develop different synthetic biology chassis systems. For now, we invite all *K. phaffii* researchers and users to contribute to, and benefit from, our OPENPichia resource.

## Methods

### Strains and media

The wild-type *K. phaffii* strains NRRL YB-4290, NRRL Y-7556 and NRRL Y-11430 were obtained from the Agricultural Research Service, CBS 2612 was obtained from the Westerdijk Institute (Netherlands) and NCYC 2543 was obtained from the National Collection of Yeast Cultures. All mentioned strains were cultured and maintained on YPD or YPD agar.

All entry and expression vectors were propagated and are available in the *E. coli* DH5α strain. MC1061 and MC1061λ strains were also successfully used and generally showed higher transformation efficiency as well as easier green–white or red–white screening than was the case for DH5α. All *E. coli* strains were cultured and maintained on Luria–Bertani (LB) agar.

The following antibiotics were used at a concentration of 50 µg ml^−1^ for the selection in *E. coli*: Zeocin, nourseothricin, hygromycin, kanamycin, chloramphenicol and carbenicillin. The following antibiotics were used at a concentration of 100 µg ml^−1^ for the selection in *K. phaffii*: Zeocin, nourseothricin, hygromycin, geneticin and blasticidin.

Several media were used: LB (1% tryptone, 0.5% yeast extract and 0.5% NaCl), yeast extract peptone dextrose (YPD; 1% yeast extract, 2% peptone and 2% d-glucose), yeast extract peptone glycerol (YPG; 1% yeast extract, 2% peptone and 1% glycerol), BMY (1% yeast extract, 2% peptone, 1.34% yeast nitrogen base without amino acids and 100 mM potassium phosphate buffer pH 6), buffered minimal glycerol yeast extract medium (BMGY; BMY with 1% glycerol), BMDY (BMY with 2% d-glucose), buffered methanol-complex medium (BMMY; BMY with 1% methanol) and limiting glucose (1% yeast extract, 2% peptone, 100 mM phosphate buffer pH 6, 50 g l^−1^ Enpresso EnPump substrate and 5 ml l^−1^ Enpresso EnPump enzyme solution). For plates, 1.5% agar was added to the LB media and 2% to the YPD media; when Zeocin selection was used, the media were set to pH 7.5.

All oligonucleotides and synthetic DNA fragments were ordered from Integrated DNA Technologies. All synthetic DNA fragments (gBlocks and Genes) were designed and adapted for synthesis using the Codon Optimization Tool and gBlocks Gene Fragments Entry Tool available at the website of Integrated DNA Technologies Europe.

### Illumina sequencing

The strains were cultured overnight in YPD medium and the genomic DNA (gDNA) was extracted using an Epicentre MasterPure Yeast DNA Purification Kit. Sample preparation (DNA fragmentation, adaptor ligation, size selection and amplification) and next-generation sequencing (5 × 10^6^ 150-bp paired-end reads) was done by Eurofins using Illumina technology. The raw sequence reads were uploaded to the NCBI database under the accession number PRJNA909165. The reads were checked for quality using fastqc^38^, from which the %GC and number of reads were obtained. From the number of reads, the average overall coverage was calculated using the formula $\frac{\mathrm{{reads}}\times {\mathrm{read}}\,{\mathrm{length}}\left({\mathrm{bp}}\right)}{{\mathrm{length}}\; {\mathrm{of}}\; {\mathrm{genomic}}\; {\mathrm{DNA}}+{\mathrm{mitochondrial}}\; {\mathrm{DNA}}\left({\mathrm{bp}}\right)}$.

#### Next-generation sequencing analysis

The reads were trimmed using Trimmomatic^39^ to remove adaptors, leading and trailing low-quality bases (cut off quality of three), low-quality reads (four-base sliding window quality of <15) and reads below 100 bp. Next, the reads were aligned to a reference and the mutations were identified using Breseq^40^ in consensus mode. The genome sequence published by Sturmberger et al.^12^ was used as a reference. The reference sequences for killer-like plasmids and the mitochondrial DNA were obtained from Sturmberger et al.^12^ and Brady et al.^16^, respectively. The reported coverage depth was calculated using the Breseq algorithm. This is done by fitting a negative binomial distribution to the read-coverage depth observed at unique reference positions. The mean of this binomial fit is used as the coverage depth. The copy number of killer-like plasmids was estimated by comparing their coverage depth with the average of the four chromosomes. The coverage depth for each molecule was calculated as the mean of a binomial fit for the coverage depth for each reference position.

#### Phylogenetic tree

To generate a phylogenetic tree, the sequencing data from this study were combined with the previously published raw reads^14^ and also aligned as described above. From the predicted mutations of both datasets, a whole genome alignment was constructed, from which a phylogenetic tree was calculated using the Mega X^41^ software package. A maximum likelihood algorithm was used with a Hasegawa–Kishino–Yano substitution matrix.

### Creation of the NCYC 2543 Δ*his4* strain

The NCYC 2543 Δ*his4* strain was generated using the split-marker method that was described previously by Heiss and colleagues^42^. The homology arms of the *HIS4* gene were selected from Näätsaari et al.^43^ and the reference genome of the CBS 7435 strain. First, a construct containing the two homology arms with a floxed nourseothricin acetyltransferase marker was created. Two overlapping fragments, which overlap for a length of 594 bp, containing one of the homologies and a part of the antibiotic marker were then generated by PCR using Taq polymerase (Promega). These fragments were purified through phenol–chloroform precipitation. Briefly, following the addition of an equal volume of phenol:chloroform:isoamyl alcohol (25:24:1), the solution was mixed, centrifuged (5 min at 12,000*g*) and the liquid phase was isolated by decanting. A one-tenth volume of 3 M sodium acetate pH 5.5 and two volumes of 100% ethanol was added the sample, which was then mixed and centrifuged (15 min at 12,000*g*). Finally, the pellet containing the amplified DNA was washed with 70% ethanol, air-dried and resuspended in water.

Both purified fragments were transformed into NCYC 2543 competent cells by electroporation, and the transformants were streaked to single clone onto YPD plates containing nourseothricin and cultured at room temperature for 2 days. The resulting clones were replica plated onto CSM-his plates for growth screening and cultured for 2 days at room temperature. Strict non-growers were checked by colony PCR for replacement of the *HIS4* gene with the antibiotic marker cassette.

The nourseothricin acetyltransferase marker was finally removed by transient expression of a *Cre*-recombinase. This gene was cloned into a plasmid with an autonomously replicating sequence^44^ and a Zeocin-resistance cassette, which was then transformed into the Δ*his4* strain. The transformants were incubated overnight on a YPD plate containing Zeocin and the resulting colonies were transferred to YPD plates without antibiotics. The removal of the antibiotic cassettes of the plasmid and *HIS4* knockout was verified with replica plating on YPD containing the respective antibiotics and double-checked via colony PCR.

### Creation of the NCYC 2543 *hoc1*^tr^ strains

The NCYC 2543 *hoc1*^tr^ strains were generated using the split-marker method described in the previous section. The left homology arm of the *HOC1* gene was chosen such that it contained about 1 kb upstream of the premature stop codon. *K. phaffii* gDNA was used as the PCR template. The right homology arm was chosen so that it contained about 1 kb downstream of the premature stop codon. The left and right homology arms were respectively fused by PCR to the first and last two-thirds of the floxed nourseothricin acetyltransferase marker. The PCR fragments were gel purified and the DNA was recovered using a Wizard SV Gel and PCR Clean-Up System (Promega) according to the manufacturer’s instructions. Both purified fragments were transformed into NCYC 2543 competent cells by electroporation, and the transformants were streaked to single clone onto YPD plates containing nourseothricin and cultured at room temperature for 2 days. The resulting clones were screened through colony PCR using a forward primer that annealed upstream of the left homology arm and a reverse primer that annealed to the nourseothricin selection marker. The nourseothricin acetyltransferase marker was removed by transient expression of a *Cre*-recombinase as described in the previous section. The engineered *HOC1* locus was confirmed for both strategies by colony PCR and Sanger sequencing. The sequences for the PCR primers and split-marker cassettes are in Supplementary Tables 6 and 7.

### Growth analysis

The different *K. phaffii* strains were cultured on YPD agar for 2 days, inoculated in triplicate into a 5 ml preculture in test tubes containing BMDY and cultured overnight at 28 °C with shaking at 225 rpm The optical density at 600 nm (OD_600_) of each culture was measured and 250 ml BMDY was inoculated at a starting OD_600_ of 0.05. Samples of 1 ml were immediately isolated from each culture to measure and check the starting OD_600_. Next, the culture was cultivated in shake flasks at 28 °C with shaking at 225 rpm; samples of 1 ml were isolated every 2 h for 22 h and again after 26 and 29 h. All samples were diluted accordingly and measured within an OD_600_ range of 0.05–1.00.

### Recombinant protein expression

The expression vectors were made using a MoClo toolkit, based on Golden Gate cloning as described in this paper (Supplementary Data 2). Briefly, the protein-coding sequences were ordered synthetically with Part 3b-type BsaI overhangs (NEB, R3733) and cloned into the entry vector with BsmBI (NEB, R0739). Next, expression vectors were made by assembly of the Level 0 parts.

The cloning procedure was as follows: 1 µl T4 DNA ligase (400 U; NEB, M0202), 2 µl T4 DNA ligase buffer (NEB, M0202) and 1 µl restriction enzyme (20 U) were added to 20 fmol backbone (pPTK081 for entry vectors or any P8 backbone for destination vectors). An excess of insert (>1,000 fmol PCR amplicon or synthetic gene, or 10 pmol annealed oligonucleotides) was added for a BsmBI assembly, whereas equimolar amounts (20 fmol) of each entry vector were added for a BsaI assembly. BsmBI assembly mixtures were incubated according to the following protocol: >25 cycles of 42 °C for 2 min (digest) and 16 °C for 5 min (ligation), followed by 60 °C for 10 min (final digest) and 80 °C for 10 min (heat inactivation step). BsaI assembly mixtures were incubated similarly, except that the digestion steps were performed at 37 °C.

*K. phaffii* electrocompetent cells were generated using the previously described lithium acetate method^45^. Briefly, precultures were inoculated in 5 ml YPD and cultured overnight in an incubator at 28 °C with rotation at 250 rpm. The precultures were diluted and cultured to an OD_600_ of approximately 1.5. Cells were harvested by centrifugation (1,519*g* for 5 min at 4 °C) from 50 ml of the culture, resuspended in 200 ml of a lithium acetate and dithiothreitol solution (100 mM lithium acetate, 10 mM dithiothreitol, 0.6 M sorbitol and 10 mM Tris–HCl pH 7.5) and incubated at 28 °C for 30 min with rotation at 100 rpm. The cells were then collected by centrifugation (1,519*g* for 5 min at 4 °C), washed twice with 1 M ice-cold sorbitol and finally resuspended in 1.875 ml of 1 M ice-cold sorbitol. DNA (0.5–1 µg) was added to aliquots of 80 µl and electroshocked (1.5 kV, 200 Ω and 25 µF). A 1 ml volume of 1 M sorbitol was immediately added to the samples and the suspension was incubated at 28 °C for 2–5 h. Next, the cells were plated on YPD agar containing the appropriate antibiotic and colonies were isolated after 2 days of incubation at 30 °C.

To enable the comparison of expression levels, only colonies with single-copy integration of the construct were selected. The copy number was determined by quantitative PCR on a LightCycler 480 system (Roche) using primers that bind PAOX1 and PGAP. The genes *OCH1* and *ALG9* were used as references. NCYC 2543 gDNA was included as a single-copy positive control. A single-copy plasmid integration will yield one additional copy and more than two copies would be the result of multiple plasmid integrations. Amplification efficiencies were determined using serial dilutions of gDNA samples. Reactions were set up in 10 μl with final concentrations of 300 nM forward primer, 300 nM reverse primer, 1×SensiFast SYBR no-ROX mastermix (Bioline), 10 ng gDNA and the following cycling conditions: 3 min at 95 °C, followed by 45 cycles of 95 °C for 3 s, 60 °C for 30 s at a ramp rate 2.5 °C s^−1^ and 72 °C for 1 s, and ending with 0.11 °C s^−1^ from 65 °C to 95 °C for melting curve determination (5 acquisitions s^−1^). Copy numbers were calculated using the ΔΔ*C*_t_ method^46^.

The different strains expressing the recombinant proteins were cultured on YPD agar plates for 2 days, inoculated in triplicate into a 5 ml preculture of BMDY and cultured at 28 °C overnight with shaking at 225 rpm. Next, the cultures for PAOX1-driven expression were inoculated in 2 ml BMDY, cultured for 24 h in a microtiter plate, transferred to 2 ml BMMY and incubated for 48 h in a microtiter plate. After 24 h in BMMY, an extra 1% methanol was added. The cultures for PGAP-driven expression were instead inoculated in 2 ml limiting glucose medium and incubated for 48 h in a microtiter plate. The OD_600_ was measured for all cultures and the supernatant was collected by centrifugation (2,500*g* for 5 min). The samples were incubated with EndoH (produced in-house) to remove *N*-glycans and analysed by SDS–PAGE.

### ELISA-based quantification of GBP

Each well of a Nunc MaxiSorp 96-well plate was coated with 75 ng anti-penta-His (Qiagen, 34660) in PBS solution and incubated overnight at 4 °C. The wells were washed three times with 200 µl wash buffer (PBS + 0.05% Tween-20) and any residual liquid was removed. The samples were blocked with 100 µl Reagent Diluent (1% Probumin (Millipore, 82-045-1) in PBS pH 7.2) for 2 h. This was followed by three washes with 200 µl wash buffer and the removal of any residual liquid. Dilutions of the yeast supernatant were prepared in 96-deep-well plates, and 100 µl of a 100,000-fold dilution was applied to each well, followed by incubation for 1 h with gentle shaking in a table-top plate shaker. The wells were washed three times with 200 µl wash buffer and the residual liquid was removed. The samples were provided with 100 µl of 250 ng ml^−1^ MonoRab rabbit anti-camelid VHH coupled to horseradish peroxidase in Reagent Diluent and incubated for 1 h with gentle shaking in a table-top plate shaker. Each well was washed three times with 200 µl wash buffer and the residual liquid was removed. 3,3′,5,5′-Tetramethylbenzidine substrate was prepared according to the manufacturer’s instructions (BD OptEIA) and 100 µl was applied to each well, followed by a 10 min incubation. Finally, 50 µl stop solution (2 N H_2_SO_4_) was added to each well and the plate was read at 450 nm using a plate reader. The absorbance units were background corrected. All strains were compared in a Kruskal–Wallis omnibus test (two-sided), followed by a pairwise (two-sided) comparison corrected with Dunn’s multiple comparison procedure.

### Flow cytometry to compare surface display of human lysozyme

Electroporation of a surface display plasmid to multiple *K. phaffii* strains (NRRL Y-11430, NCYC 2543 and OPENPichia) was performed using the lithium acetate method described in the ‘Recombinant protein expression’ section. We chose the previously reported^47^ pPSD-FLAG-hLYZ-V5-Sag1 plasmid as a test case. It expresses the wild-type human lysozyme protein flanked by an N-terminal FLAG tag and a C-terminal V5 tag, and is fused at the C-terminal end to a Sag1 anchor under the control of the *AOX1* promoter. The copy number of the surface display construct in the resulting strains was determined as described earlier. Clones that were determined to have one integrated copy of the surface display construct were inoculated in BMGY supplemented with 50 µg ml^−1^ Zeocin in technical triplicates and cultured for 24 h at 28 °C with shaking at 200 rpm. The cultures were then transferred to BMMY supplemented with 50 µg ml^−1^ Zeocin, set to 10 OD_600_ units ml^−1^ and further cultured at 28 °C for 24 h with shaking at 200 rpm. After 12 h, the cultures were spiked with an additional 1% methanol. After induction, the cells were harvested by centrifugation at 1,500*g* for 5 min and washed three times with ice-cold washing buffer (PBS containing 1 mM EDTA pH 7.2 and one cOmplete Inhibitor EDTA-free tablet (Roche) per 50 ml buffer). The cells were kept on ice during the entire staining procedure. Unstained controls, single-stain controls and an empty vector control were included.

The cells (at an OD_600_ of two) were stained with mouse monoclonal anti-V5 (1/500; AbD Serotec, MCA2892) and rabbit polyclonal anti-FLAG (1/200; Sigma-Aldrich, F7425) in ice-cold staining buffer (wash buffer containing 0.5 mg ml^−1^ BSA) for 1 h at 4 °C. They were then washed three times with ice-cold staining buffer and stained with goat anti-mouse AF568 (1/250; Thermo Fischer Scientific, A-11031), goat anti-rabbit AF488 (1/500; Thermo Fischer Scientific, A11008) and Live/Death stain eFluor506 (1/1,000; Thermo Fischer Scientific) for 1 h at 4 °C. This was followed by three washes with ice-cold staining buffer before analysis on a BD FACSMelody instrument. The data were analysed using the FlowJo software. The gating strategy is shown in Extended Data Fig. 6.

### Comparison of NCYC 2543 and NCYC 2543 *hoc1*^tr^ in a fed-batch process

Fermentations were conducted using a SciVario Twin 3 l fermenter (Eppendorf) containing 800 ml basal salts medium as described in the *Pichia* Fermentation Process Guidelines (Invitrogen Corporation, 2002). Yeast extract (Neogen, NCM0218A) was further added at a concentration of 10 g l^−1^ to supplement the batch medium.

To prepare the inoculum seed culture, a 1 l baffled flask containing 100 ml of BMGY, 1% yeast extract, 2% peptone, 1.34% yeast nitrogen base, 1% glycerol, 100 mM potassium phosphate pH 6.0), supplemented with 4 × 10–5% biotin, was inoculated with the expression clone of interest at an initial OD_600_ of 0.1. The culture in the flask was incubated at 28 °C with agitation at 200 rpm for 20–24 h until the OD_600_ reached the range of 20–30.

The batch phase of the fermenter was initiated by inoculating batch medium with inoculum seed at an initial OD of one. The cultivation temperature was maintained at 25 °C with an airflow rate of 1 vvm. The pH was automatically controlled at 6.0 by the addition of 25% wt/wt ammonium hydroxide as required. The dissolved oxygen levels were maintained at 30% saturation through control of agitation (600–1,200 rpm) and the addition of pure oxygen. Foam formation was prevented by the addition of an antifoam solution (Struktol, J673A).

Once the initial glycerol (40 g l^−1^) was fully consumed, marked by a rapid increase in the percentage of dissolved oxygen, the fed-batch phase commenced with the introduction of a 50% glucose solution (wt/wt) supplemented with 12 ml l^−1^ PTM1 solution. The feed rate was adjusted to 20 ml h^−1^ l^−1^ batch volume and linearly increased to 40 ml h^−1^ l^−1^ batch volume over a duration of 48 h to introduce 1 l of feed solution. All process parameters were maintained at the levels established during the batch phase throughout the entire fermentation process.

### RT–qPCR analysis of *HOC1* mRNA

The four strains were inoculated in BMGY medium, in triplicate, from an overnight preculture and cultured for 20 h at 28 °C and 200 rpm. The cells (10 OD_600_ units) were pelleted and washed with RNase-free water. Total RNA was prepared using a RiboPure-Yeast Kit (Invitrogen, AM1926), followed by a DNase treatment using a TURBO DNA-free Kit (Invitrogen, AM1907) according to the manufacturer’s instructions. Complementary DNA was then prepared using an iScript cDNA Synthesis Kit (BioRad, 1708891). The RT–qPCR reaction was performed for technical triplicates of each biological replicate using the following conditions: activation for 5 min at 95 °C, followed by 40 cycles of 10 s at 95 °C, 15 s at 55 °C and 20 s at 72 °C, and a final elongation step for 40 s at 72 °C. The transcript level variance of eight reference genes for normalization (*UCB6*, *TDH3*, *QCR9*, *ALG9*, *PGK1*, *TAF10*, *ACT1* and *TPI1*) was analysed using the geNorm algorithm, as implemented in the qbase+ software^48^, to identify the genes whose transcript levels were least affected under the experimental conditions used. Based on these data (not shown), the *HOC1* transcript levels were normalized using the geometric mean of the genes *QCR9* and *ALG9*. The levels of *HOC1* transcript were determined using two primer pairs. Determination of amplification efficiencies and conversion of raw *C*_q_ values to calibrated normalized relative quantity was performed using the qbase+ software. Statistical analysis of the calibrated normalized relative quantities was done using the GraphPad Prism 9 software package. All primers used are listed in Supplementary Table 6.

### Transformation efficiency testing

Competent cells were prepared using the lithium acetate method described in the ‘Recombinant protein expression’ section. Each strain was transformed with 200 ng linearized plasmid and several dilutions of the transformation mix were plated on either non-selective YPD agar or YPD agar containing 100 µg ml^−1^ Zeocin. For each transformation, colonies were counted from the plates where clear individual colonies could be observed after incubation at 30 °C for 2 days. Both the selective and non-selective plates were counted to correct for a potential difference in the number of competent cells per transformation.

A linear model (estimated using ordinary least squares) was fitted in the statistical software R^49^. The log-transformed normalized transformation efficiency (natural logarithm of the number of transformants per million clones) was used as the outcome variable, and the strain and promotor type, including an interaction effect were used as the predictor variables. The model explains a statistically significant and substantial proportion of variance (coefficient of multiple correlation (*R*^2^) = 0.94, *F*(7,38) = 81.33, *P* < 0.001 and adjusted *R*^2^ = 0.93). Model-predicted group means with 95% confidence intervals were obtained using the ggeffects package with heteroscedasticity-consistent variance estimators from the sandwich package (vcovHC, type HC0)^50,51^.

### Capillary gel electrophoresis-laser induced fluorescence detection-based glycan analysis of cell-wall mannoproteins

Strains were inoculated in YPD or YPG medium, from their respective precultures, at an OD_600_ of 0.05 and cultured overnight at 28 °C and 200 rpm. The next day, 500 OD_600_ units per strain were pelleted (10 min at 1,500*g*) and the mannoproteins were isolated as follows. The pellets were washed three times with Milli-Q water, after which 20 mM citrate buffer pH 6.6 was added at 1 ml per 150 µg of wet cell weight. The resuspended cells were autoclaved for 1.5 h at 120 °C in cryovials and then centrifuged for 10 min at 16,000*g*. Three volumes of ice-cold methanol were added to the supernatant fractions and the vials were incubated for 15 min at 20 °C. The mannoproteins were spun down at 16,000*g* for 10 min and the pellets were left to dry until transparent. The pellets were resuspended in 50 µl RCM buffer (8 M urea, 360 mM Tris–HCl pH 8.6 and 3.2 mM EDTA) and stored at 4 °C until further analysis.

*N*-linked oligosaccharides were prepared from the purified mannoproteins following blotting to polyvinylidene fluoride membrane in the wells of 96-well plate membrane plates and analysed by capillary electrophoresis with laser-induced fluorescence detection using an ABI 3130 capillary DNA sequencer as described previously^22^.

### Alcian blue assay

The assay was performed as described previously^23^, with the following adaptations. Briefly, Alcian blue was prepared in 0.02 N HCl at a concentration of 63 µg ml^−1^ and the solution was centrifuged to remove insoluble precipitates. An overnight culture of each strain was cultured in YPD medium at 28 °C and 200 rpm. The next day, the cells were pelleted and the supernatant was removed. The cells were washed with 0.02 N HCl and the pellet was resuspended in 0.02 N HCl to 10 OD_600_ units ml^−1^. The cells (100 µl; 1 OD_600_) were transferred to a 96-well V-bottomed plate, to which 100 µl of the Alcian blue solution was added. Following incubation at room temperature for 15 min, the plate was centrifuged at 3,220*g* for 15 min, after which the pellets were visually checked.

### Congo red and Calcofluor white test

The test was performed as described elsewhere^52^, with slight adaptations. Briefly, the strains were cultured overnight in BMGY. The next day, dilutions were made to obtain between 1 × 10^5^ and 10 cells in 5 µl BMGY. Drops of 5 µl were spotted on the different plates, which were incubated at 30 °C for 3 days. Congo red (Sigma, C6767) and Calcofluor white (Fluka, 18909) were present at final concentrations of 75 µg ml^−1^ and 10 µg ml^−1^, respectively.

### Electron microscopy

#### Transmission electron microscopy

The strains were cultured overnight in BMGY at 28 °C and 200 rpm. High-pressure freezing, as described previously^53^, was carried out in a high-pressure freezer (Leica EM ICE). The cells were pelleted and frozen as a paste in 150 µm copper carriers. High-pressure freezing was followed by quick freeze substitution as described previously^54^. Briefly, the carriers were placed on top of the frozen FS solution inside a cryovial containing 1% double-distilled water, 1% OsO_4_ and 0.5% glutaraldehyde in dried acetone. After reaching 4 °C for 30 min, the samples were infiltrated stepwise over 3 days at 0–4 °C in Spurr’s resin and embedded in capsules. The polymerization was performed at 70 °C for 16 h. Ultrathin sections of a gold interference colour were cut using an ultramicrotome (Leica EM UC6), followed by post staining, in a Leica EM AC20 system, with uranyl acetate at 20 °C for 40 min and lead at 20 °C for 10 min.

The sections were collected on formvar-coated copper slot grids. The grids were viewed using a JEM-1400Plus transmission electron microscope (JEOL) operating at 60 kV.

#### Scanning electron microscopy

The strains were cultured overnight in BMGY at 28 °C and 200 rpm. The cells were fixed overnight in 1.5% paraformaldehyde and 3% glutaraldehyde in 0.05 M sodium cacodylate buffer pH 7.4. The fixed cells were centrifuged for 2 min at 1,000*g* between each of the following steps. First, the cells were washed three times with 0.1 M sodium cacodylate buffer pH 7.4 and then incubated for 30 min in 0.1 M sodium cacodylate pH 7.4 containing 2% OsO_4_. The osmicated samples were washed three times with Milli-Q water before a stepwise ethanol dehydration (50%, 70%, 90% and 2 × 100%). This was followed by two incubations in hexamethyldisilazane solution (Sigma-Aldrich), as a final dehydration step, after which the samples were spotted on silicon grids (Ted Pella) and air-dried overnight at room temperature. Finally, the samples were coated with 5 nm platinum in a Q150T ES sputter coater (Quorum Technologies) and placed in a Gemini 2 Cross beam 540 microscope (Zeiss) for scanning electron microscopy imaging at 1.50 kV using an SE2 detector.

### Reporting summary

Further information on research design is available in the Nature Portfolio Reporting Summary linked to this article.

### Supplementary information

Supplementary Information Supplementary Tables 1–7.

Reporting Summary

Supplementary Table Supplementary Tables 1–7.

Supplementary Data 1 Sanger sequencing of the *hoc1*^tr^ region.

Supplementary Data 2 FASTA files of all MoClo Parts.

Supplementary Data 3 GenBank file of all model proteins.

### Source data

Source Data Fig. 2 Source data.

Source Data Fig. 3 Source data.

Source Data Fig. 5 Unprocessed SDS–PAGE gels for Fig. 5a and source data of Fig. 5b.

Source Data Extended Data Fig. 1 Source data.

Source Data Extended Data Fig. 2 Source data.

Source Data Extended Data Fig. 7 Unprocessed SDS–PAGE gels.

## Data Availability

All raw reads of the genomes sequenced in this study have been submitted to the NCBI and can be found under the accession number PRJNA909165. The CBS 7435 reference genome can be found under the NCBI accession number GCA_900235035.2. Source data are provided. All other data supporting the findings of this study are available from the corresponding authors. OPENPichia is available from VIB (OPENPichia.com). The expression vector construction toolkit can be obtained from the BCCM at https://bccm.belspo.be/catalogues/plasmid-sets/openpichia.
