# Supplementary material for: OPENPichia: licence-free Komagataella phaffii chassis strains and toolkit for protein expression
Source: Nat Microbiol. 2024 Mar 4;9(3):864–76. doi: 10.1038/s41564-023-01574-w (PMC10914597; doi:10.1038/s41564-023-01574-w)
Supplement: Supplementary file 1 — Supplementary Tables 1–7. [file 41564_2023_1574_MOESM1_ESM.pdf]

nature microbiology

Resource

https://doi.org/10.1038/s41564-023-01574-w

OPENPichia: licence-free *Komagataella phaffii* chassis strains and toolkit for protein expression

In the format provided by the authors and unedited

Supplementary information

Table of Contents

Supplementary Table 1	Page 2
Supplementary Table 2	Page 2
Supplementary Table 3	Page 2
Supplementary Table 4	Page 3
Supplementary Table 5	Page 3
Supplementary Table 6	Page 3
Supplementary Table 7	Page 5

Tables

Supplementary Table 1. Strains used in this publication. * Type strain; Abbreviations: UCD: University of California, Davis, USA; CBS: Centraalbureau voor Schimmelculturen, currently known as Westerdijk Fungal Biodiversity Institute, The Netherlands; NCYC: National Collection of Yeast Cultures, UK; NRRL: Northern Regional Research Laboratory, currently known as Agricultural Research Service (ARS), USA; NTG: nitrosoguanidine.

Strain ID	Origin of strain isolate	Source	Original depositor
CBS 2612 *	*Quercus kelloggii* (California, USA)	CBS culture collection	HJ Phaff, UCD
NCYC 2543 *	*Quercus kelloggii* (California, USA)	NCYC culture collection	CBS
NRRL YB-4290 *	*Quercus kelloggii* (California, USA)	NRRL culture collection	HJ Phaff, UCD
NRRL Y-7556 *	*Quercus kelloggii* (California, USA)	NRRL culture collection	D. Yarrow, CBS
UCD FST K-239 *	*Quercus kelloggii* (California, USA)	UCD culture collection	HJ Phaff, UCD
NRRL Y-11430	Most likely a subclone of the type strain	NRRL culture collection	Patent deposit Phillips Petroleum Company (USA)
GS115	NTG-mutagenized derivative of NRRL Y-11430	Invitrogen	
NCYC 2543 *his4*	Genetic engineering	This publication	
NCYC 2543 *hoc1^{tr}-1*	Genetic engineering	This publication	
NCYC 2543 *hoc1^{tr}-2*	Genetic engineering	This publication	

Supplementary Table 2. Overview of the next generation sequencing results. For each sequenced strain, the NCBI accession number of the raw reads, the total number of reads, %GC and average overall coverage are reported.

Strain Designation	NCBI accession number	Number of reads	%GC	Average overall coverage
NRRL Y-11430	SRX18507532	11883858	37	191
NRRL YB-4290	SRX18507533	16291010	31	261
NCYC 2543	SRX18507534	14748310	37	236
CBS 2612	SRX18507535	13586250	39	218
NRRL Y-7556	SRX18507536	13886574	32	223
NCYC 2543 *hoc1^{tr}-1*	SRX19450313	10952566	37	175
NCYC 2543 *hoc1^{tr}-2*	SRX19450314	8082342	37	129

Supplementary Table 3. Proportion of NGS reads mapping to different molecules of the reference genome, mitochondrial DNA and killer-like plasmids.

Strain Designation	Reads mapped to		
	Genome (%)	Mitochondrial DNA (%)	Killer-like plasmids (%)
NRRL Y-11430	79.6	17.7	2.7
NRRL YB-4290	45.1	47.3	7.7
NCYC 2543	78.5	21.6	0
CBS 2612	89.4	10.7	0
NRRL Y-7556	52.4	38.7	9
NCYC 2543 *hoc1^{tr}-1*	82.7	17.3	0
NCYC 2543 *hoc1^{tr}-2*	80.8	19.2	0

Supplementary Table 4. Overview of the functional mutations of the analysed *K. phaffii* strains compared to the CBS 7435 (eq. strain deposit of NRRL Y-11430) reference genome, non-functional SNPs and indels are between brackets. Functional mutations are those resulting in a SNP or indel in a coding sequence.

Strain Designation	SNP	Indel	Total	Mutations/Mbp	Killer-like plasmids copy number (KP1/KP2)
NRRL Y-11430	0 (2)	0 (19)	0 (21)	2.3	21/15
NRRL YB-4290	2 (2)	1 (20)	3 (22)	2.9	140/82
NCYC 2543	3 (2)	1 (22)	4 (24)	3.1	none detected
CBS 2612	4 (3)	1 (25)	5 (28)	3.1	none detected
NRRL Y-7556	3 (2)	1 (21)	4 (23)	3.2	138/86
NCYC 2543 *hoc1*^{tr}*-1*	3 (2)	1* (21)	4 (23)	3.5	none detected
NCYC 2543 *hoc1*^{tr}*-2*	3 (2)	1** (20)	4 (22)	3.3	none detected

*: Equal to NRRL Y-11430 i.e. $T_{5\rightarrow6}$, but LoxP site integrated

**: Equal to NRRL Y-11430 i.e. $T_{5\rightarrow6}$, but 115bp deletion and LoxP site integrated

Supplementary Table 5. Details of the selected proteins used for the protein expression comparisons.

Abbreviation	Name	Type	Molecular mass (kDa)	Secretion leader	Ref.
GM-CSF	Granulocyte-macrophage colony-stimulating factor	Cytokine	15.7	αMF	[61]
GaOx	Galactose oxidase from *Fusarium graminearum*	Enzyme	69.9	αMF	[62]
Cdiff-VHH-IgA	Anti-*C.difficile* toxin VHH IgA fusion protein	VHH-IgA	41.2	Ost1	[63]
CovidVHH-IgG	SARS-CoV-2 neutralizing VHH hIgG1 fusion protein	VHH-IgG	40.6	αMF	[64]
GBP	GFP-binding protein	VHH	14.1	αMF	[65]

Supplementary Table 6. List of oligonucleotides that were used as primers for PCR, cPCR, RT-qPCR or sequencing.

Primer name	Sequence (5' → 3')	Used for
OCH1_Fw	CCTCTGATAGTTCCTTTCCG	Fw primer for qPCR (copy number determination) for reference gene *OCH1*
OCH1_Rv	AAGACTTCTGGTACACGTTC	Rev primer for qPCR (copy number determination) for reference gene *OCH1*
ALG9_Fw	CTTTAGTGGGATGTTACCAG	Fw primer for qPCR (copy number determination) for reference gene *ALG9*
ALG9_Rv	CAACGTAAAAATCACACTCC	Rev primer for qPCR (copy number determination) for reference gene *ALG9*
GAPp_Fw	CCGGGGTAAAACTTAAATGTG	Fw primer for qPCR (copy number determination) for *GAP* promoter
GAPp_Rv	GGGTACACGACCTCCGTTTT	Rev primer for qPCR (copy number determination) for *GAP* promoter
AOX1p_Fw	CCATCCGACATCCACAGGTC	Fw primer for qPCR (copy number determination) for *AOX1* promoter
AOX1p_Rv	TCGATGGCAAAAGTGGGTGT	Rev primer for qPCR (copy number determination) for *AOX1* promoter

AOX1tt_Fw	TGCCATTTGCCTGAGAGATG	Fw primer for qPCR (copy number determination) for *AOX1* transcription terminator
AOX1tt_Rv	TTCCCAAACCCCTACCACAAG	Rev primer for qPCR (copy number determination) for *AOX1* transcription terminator
72_HOC1_1a_Fw	GAGTGTCGCACGTCTCGG	Fw primer left homology arm *HOC1* (strategy 1&2)
73_HOC1_1b_Rev	ATGCTATACGAACGGTATCAAGATTGGGTGACCAGGC	Rev primer left homology arm *HOC1* – overlap with Nour selection marker (strategy 1&2)
74_HOC1_2a_Fw	CATTATACGAACGGTAGAGAATTAATCGCCAAAATCACAGAGG	Fw primer right homology arm *HOC1* – overlap with Nour selection marker (strategy 1)
75_HOC1_2b_Rev	GGATTCAATCCCGGATTTGTCG	Rev primer right homology arm *HOC1* (strategy 1&2)
76_Lox71_3a_Fw	CACCCAATCTTGATACCGTTCGTATAGCATACATTATACG	Fw primer first two thirds of Nour selection marker – overlap with left homology arm *HOC1* (strategy 1&2)
77_2/3Nour_3b_Rev	CAACGGTCAACCTTCTATTCC	Rev primer first two thirds of Nour selection marker (strategy 1&2)
78_2/3Nour_4a_Fw	CTCGAAACCGCGACTTC	Fw primer second two thirds of Nour selection marker (strategy 1&2)
79_Lox66_4b_Rev	GCGATTAATTCTCTACCGTTCGTATAATGTATGCTATACG	Rev primer second two thirds of Nour selection marker – overlap with right homology arm *HOC1* (strategy 1)
82_HOC1_2c_Fw	CATTATACGAACGGTAGCAATGGACGGGTACTGG	Fw primer right homology arm *HOC1* – overlap with Nour selection marker (strategy 2)
83_Lox66_4c_Rev	CCGTCCATTGCTACCGTTCGTATAATGTATGCTATACG	Rev primer second two thirds of Nour selection marker – overlap with right homology arm *HOC1* (strategy 2)
11_US_HOC1_Fw	GGGGTTCGATAATCAAACTTGCCTTGAAGG	Colony PCR primer upstream of left homology arm of *HOC1*
12_DS_HOC1_Rev	CACCGATACCCAGACCGTATCAACCAC	Colony PCR primer downstream of right homology arm of *HOC1*
QCR9_Fw	AGAAACTCTACTTATGTTGCC	RT-qPCR primer for the *QCR9* reference gene (RNA transcription)
QCR9_Rv	CTTTCCTCTGTTATGAGCTT	RT-qPCR primer for the *QCR9* reference gene
ALG9_Fw	CTTTAGTGGGATGTTACCAG	RT-qPCR primer for the *ALG9* reference gene
ALG9_Rv	CAACGTAAAAATCACACTCC	RT-qPCR primer for the *ALG9* reference gene
106_qPCR1_HOC1_Fw	ATTTGGCTCACGGTTGGACT	RT-qPCR primer for *HOC1*
107_qPCR1_HOC1_Rev	TTTCTGAAGGTCTGTGGCGG	RT-qPCR primer for *HOC1*
108_qPCR2_HOC1_Fw	TACGCCAAGGGAGGTGTCTA	RT-qPCR primer for *HOC1*
109_qPCR2_HOC1_Rev	CCCGATGCTTTTTGGGGAGA	RT-qPCR primer for *HOC1*

Supplementary Table 7. Sequences of the split-marker fragments used to generate the two *HOC1* mutants.

Fragment	Sequence (5' → 3')
HOC1 left fragment – strategy 1&2	gagtgtcgcacgtctcggtatttttttacttgtcgattctctcttactgtccaagatgtacgaaagatgtgaagtttctttgttggcga<br>ttttcttgcagcgcgatcgcggtcggtatgatttcccttcagaccaaaaacagatatagatattcaattgaaactcgcccttccaacac<br>cctgaacaccacccccctaatgcaacaacagctgtttagaaaagtaatttggctcacggttggactcataacggtgatattggtaatаat<br>aaagatttcatccagcaagtccaccgccacagaccttcagaaagtattgaagaatgccaatattttaccgcaggacgtgatcaattaca<br>atagcagaaaagtaacggatgaactcgcttcaaagctggacgagattcaaaagaagtatctctcgaagcaagatgatagaattagcaag<br>ctcgaagctgaacgggcagatctactggaacaggttagatttctaaggaacccccctgcaggatcaagtttaagagaaaaattggctta<br>tctgtttccttataatgaaaacggcaaattccctgcttatatatggcaaacatggaagtatggcttgaatgacgatcggtttggagaaa<br>agttcaaagaaggcgaaactcagtgggcttcgaagaatcctggtttcgttcatgagttgtttaacgatgatacttccggtgtgtttatt<br>caccatctgtatatcaatgttccagaagtgatcaaagcatacgagctgcttcccaacataatcttgaaaatggacttcttcagatattt<br>ggttttatacgccaagggaggtgtctatgcagacgttgatactatgcctcttcagcctgtaccaaactggattcctgaaaatgtctccc<br>caaaaagcatcgggatgatcattggaatacaaaacgatgctaacaatccagattggaaaaagattacgtacatcgtctacaattttcca<br>attggtgtattcaagcgaagcctggtcacccaatcttgataccgttcgtatagcatacattatacgaagttatatccttcagtaatgtc<br>ttgtttcttttgttgcagtggtgagccattttgacttcgtgaaagtttctttagaatagttgtttccagaggccaaacattccacccgt<br>agtaaagtgcaagcgtaggagaaccaagactggcataaatcaggtataagtgtcgagcactggcaggtgatcttctgaaagtttctact<br>agcagataagatccagtagtcatgcatatggcaacaatgtaccgtgtggatctaagaacgcgtcctactaaccttcgcattcgttggtc<br>cagtttgttgttatcgatcaacgtgacaaggttgtcgattccgcgtaagcatgcatacccaaggacgcctgttgcaattccaagtgagc<br>cagttccaacaatctttgtaatattagagcacttcattgtgttgcgcttgaaagtaaaatgcgaacaaattaagagataatctcgaaac<br>cgcgacttcaaacgccaatatgatgtgcggcacacaataagcgttcatatccgctgggtgactttctcgctttaaaaaattatccgaaa<br>aaatttctagagtgttgttactttatacttccggctcgtataatacgacaaggtgtaaggaggactaaaccatgggtactaccttaga<br>tgatacagcctacagatacagaacatcagtccctggtgatgctgaagcaattgaggctttagacggttcattcaccaccgacaccgtct<br>ttagagtaaccgccaccggtgatggatttaccttaagagaagtcccagtcgaccctccattaactaaagtctttccagatgatgaatct<br>gatgacgaaagcgacgacggagaagatggtgacccagattcaagaactttcgtagcatacggtgatgacggtgatttggctggttttgt<br>agtcgtttcttattcaggttggaatagaaggttgaccgttg
HOC1 right fragment – strategy 1	ctcgaaaccgcgacttcaaacgccaatatgatgtgcggcacacaataagcgttcatatccgctgggtgactttctcgctttaaaaaatt<br>atccgaaaaaatttctagagtgttgttactttatacttccggctcgtataatacgacaaggtgtaaggaggactaaaccatgggtact<br>accttagatgatacagcctacagatacagaacatcagtccctggtgatgctgaagcaattgaggctttagacggttcattcaccaccga<br>caccgtctttagagtaaccgccaccggtgatggatttaccttaagagaagtcccagtcgaccctccattaactaaagtctttccagatg<br>atgaatctgatgacgaaagcgacgacggagaagatggtgacccagattcaagaactttcgtagcatacggtgatgacggtgatttggct<br>ggttttgtagtcgtttcttattcaggttggaatagaaggttgaccgttgaagatatagaagtcgccccagagcatagaggtcatggtgt<br>aggaagagctttgatgggtttggctacagaatttgcaagagagagaggagccggtcatttatggttagaagttactaatgttaacgccc<br>ctgctatccatgcttatagaagaatgggtttcacattatgtggtttagatactgctttatatgatggaacagcatctgacggtgaacag<br>gccttgtatatgtctatgccttgcccttaagccgagtaactgacaataaaaagattcttgttttcaagaacttgtcatttgtatagttt<br>ttttatattgtagttgttctattttaatcaaatgttagcgtgatttatattttttttcgcctcgacatcatctgcccagatgcgaagtt<br>aagtgcgcagaaagtaatatcatgcgtcaatcgtatgtgaatgctggtcgctatactgataacttcgtatagcatacattatacgaacg<br>gtagagaattaatcgccaaaatcacagaggatacactgcaacgagccgagtcaaactcactggaactagctgacattagcgaagaaggc<br>ggcctgtctgataagaatttgtccattatgcaatggacgggtactggtatttttacagatgccatatttacctattttaatgactacat<br>tcaaagtagtatctataccaaagttacttggaaagaattctccaattgagaaagcccaagcttgtcagtgatgtattggtactgccga<br>ttatcagcttctcggccggtgcaggtagtggaaaatcgactgaactgaacgatcccttagcattcgtacaacattatttgaaagatta<br>cataacgacaaccactaagggtcagaaccatttagattgtctggatctatcattatggccttgtttatagacaaagaattgtatcctgg<br>actgaagggaagtttatagagtaatacccctctgacaaccaactcgaatgggtattttgagataatttcccatatattattgtcttcac<br>tgggagacttacttcttctttgtagttccggtatcggcagcaaacttatggtggtcacatcttccaaacttctcggtatagtagaacta<br>tttgtggttgatacggtctgggtatcggtgataatgcttgtaataatggattttgtgggtgtaactgaagctggtgtattagtggtagt<br>ggtagtggtggttgttactgtgctatctgtaggttctgggagtggtttctgagccgggtttaaaattttgatctctgtggtatcttcgt<br>ctggatttcgactaaaaaatccgccttgaaatacgagtggaacaggtaaacgattgatggagcccagaagcactgtgtccaaggaagat<br>tcatcaaaagtgaaaggagtgccatcttcgtttccatcgttaagatgagaacttttggcaaaaaaatcaatcagaacgtcatctatttc<br>tatggcgacaaatccgggattgaatcc
HOC1 right fragment – strategy 2	ctcgaaaccgcgacttcaaacgccaatatgatgtgcggcacacaataagcgttcatatccgctgggtgactttctcgctttaaaaaatt<br>atccgaaaaaatttctagagtgttgttactttatacttccggctcgtataatacgacaaggtgtaaggaggactaaaccatgggtact<br>accttagatgatacagcctacagatacagaacatcagtccctggtgatgctgaagcaattgaggctttagacggttcattcaccaccga<br>caccgtctttagagtaaccgccaccggtgatggatttaccttaagagaagtcccagtcgaccctccattaactaaagtctttccagatg<br>atgaatctgatgacgaaagcgacgacggagaagatggtgacccagattcaagaactttcgtagcatacggtgatgacggtgatttggct<br>ggttttgtagtcgtttcttattcaggttggaatagaaggttgaccgttgaagatatagaagtcgccccagagcatagaggtcatggtgt<br>aggaagagctttgatgggtttggctacagaatttgcaagagagagaggagccggtcatttatggttagaagttactaatgttaacgccc<br>ctgctatccatgcttatagaagaatgggtttcacattatgtggtttagatactgctttatatgatggaacagcatctgacggtgaacag<br>gccttgtatatgtctatgccttgcccttaagccgagtaactgacaataaaaagattcttgttttcaagaacttgtcatttgtatagttt<br>ttttatattgtagttgttctattttaatcaaatgttagcgtgatttatattttttttcgcctcgacatcatctgcccagatgcgaagtt<br>aagtgcgcagaaagtaatatcatgcgtcaatcgtatgtgaatgctggtcgctatactgataacttcgtatagcatacattatacgaacg<br>gtagcaatggacgggtactggtatttttacagatgccatatttacctattttaatgactacattcaaagtagtatctataccaaagtta<br>cttggaaagaattctccaattgagaaagcccaagcttgtcagtgatgtattggtactgccgattatcagcttctcggccggtgcaggt<br>agtggaaaatcgactgaactgaacgatcccttagcattcgtacaacattatttgaaagattacataacgacaaccactaagggtcaga<br>accatttagattgtctggatctatcattatggccttgtttatagacaaagaattgtatcctggactgaagggaagtttatagagtaata<br>cccctctgacaaccaactcgaatgggtattttgagataatttcccatatattattgtcttcactgggagacttacttcttctttgtagt<br>tccggtatcggcagcaaacttatggtggtcacatcttccaaacttctcggtatagtagaactatttgtggttgatacggtctgggtatc<br>ggtgataatgcttgtaataatggattttgtgggtgtaactgaagctggtgtattagtggtagtggtagtggtggttgttactgtgctat<br>ctgtaggttctgggagtggtttctgagccgggtttaaaattttgatctctgtggtatcttcgtctggatttcgactaaaaaatccgcct<br>tgaaatacgagtggaacaggtaaacgattgatggagcccagaagcactgtgtccaaggaagattcatcaaaagtgaaaggagtgccatc<br>ttcgtttccatcgttaagatgagaacttttggcaaaaaaatcaatcagaacgtcatctatttctatggcgacaaatccgggattgaatc<br>c
