# Supplementary material for: OPENPichia: licence-free Komagataella phaffii chassis strains and toolkit for protein expression
Source: Nat Microbiol. 2024 Mar 4;9(3):864–76. doi: 10.1038/s41564-023-01574-w (PMC10914597; doi:10.1038/s41564-023-01574-w)
Supplement: Supplementary file 2 — Reporting Summary [file 41564_2023_1574_MOESM2_ESM.pdf]

nature portfolio

Corresponding author(s): Nico Callewaert
Katrien Claes

Last updated by author(s): Nov 24, 2023

Reporting Summary

Nature Portfolio wishes to improve the reproducibility of the work that we publish. This form provides structure for consistency and transparency in reporting. For further information on Nature Portfolio policies, see our Editorial Policies and the Editorial Policy Checklist.

Statistics

For all statistical analyses, confirm that the following items are present in the figure legend, table legend, main text, or Methods section.

n/a	Confirmed	
☐	☒	The exact sample size (*n*) for each experimental group/condition, given as a discrete number and unit of measurement
☐	☒	A statement on whether measurements were taken from distinct samples or whether the same sample was measured repeatedly
☐	☒	The statistical test(s) used AND whether they are one- or two-sided<br>*Only common tests should be described solely by name; describe more complex techniques in the Methods section.*
☐	☒	A description of all covariates tested
☐	☒	A description of any assumptions or corrections, such as tests of normality and adjustment for multiple comparisons
☐	☒	A full description of the statistical parameters including central tendency (e.g. means) or other basic estimates (e.g. regression coefficient) AND variation (e.g. standard deviation) or associated estimates of uncertainty (e.g. confidence intervals)
☐	☒	For null hypothesis testing, the test statistic (e.g. *F*, *t*, *r*) with confidence intervals, effect sizes, degrees of freedom and *P* value noted<br>*Give P values as exact values whenever suitable.*
☒	☐	For Bayesian analysis, information on the choice of priors and Markov chain Monte Carlo settings
☒	☐	For hierarchical and complex designs, identification of the appropriate level for tests and full reporting of outcomes
☒	☐	Estimates of effect sizes (e.g. Cohen's *d*, Pearson's *r*), indicating how they were calculated

Our web collection on statistics for biologists contains articles on many of the points above.

Software and code

Policy information about availability of computer code

Data collection	No software or code was used for data collection.
Data analysis	- MegaX (V1.0) software was used for the generation of a phylogenetic tree (Kumar, S., Stecher, G., Li, M., Knyaz, C. & Tamura, K. MEGA X: Molecular Evolutionary Genetics Analysis across Computing Platforms. Molecular Biology and Evolution 35, 1547–1549 (2018).)<br>- R (V4-3) and GraphPad Prism (V9) were used for statistical analysis and graph generation.<br>- FlowJo software (V10.9) was used for FACS analysis.<br>- qbase+ (V) software was used for qPCR data analysis.

For manuscripts utilizing custom algorithms or software that are central to the research but not yet described in published literature, software must be made available to editors and reviewers. We strongly encourage code deposition in a community repository (e.g. GitHub). See the Nature Portfolio guidelines for submitting code & software for further information.

Data

Policy information about availability of data

All manuscripts must include a data availability statement. This statement should provide the following information, where applicable:

- Accession codes, unique identifiers, or web links for publicly available datasets
- A description of any restrictions on data availability
- For clinical datasets or third party data, please ensure that the statement adheres to our policy

- Reference genome CBS 7435: NCBI accession code: GCA_900235035.2
- The data that support the findings of this study are available in the Source Data Files or from the corresponding authors.
- OPENPichia is available from VIB (OPENPichia.com).
- The expression vector construction toolkit can be obtained from the Belgian Coordinated Collection of Microorganisms (see https://bccm.belspo.be/catalogues/plasmid-sets/openpichia).
- NGS data for all characterized strains: NCBI accession code PRJNA909165

Research involving human participants, their data, or biological material

Policy information about studies with human participants or human data. See also policy information about sex, gender (identity/presentation), and sexual orientation and race, ethnicity and racism.

Reporting on sex and gender	NA
Reporting on race, ethnicity, or other socially relevant groupings	NA
Population characteristics	NA
Recruitment	NA
Ethics oversight	NA

Note that full information on the approval of the study protocol must also be provided in the manuscript.

Field-specific reporting

Please select the one below that is the best fit for your research. If you are not sure, read the appropriate sections before making your selection.

☒ Life sciences ☐ Behavioural & social sciences ☐ Ecological, evolutionary & environmental sciences

For a reference copy of the document with all sections, see nature.com/documents/nr-reporting-summary-flat.pdf

Life sciences study design

All studies must disclose on these points even when the disclosure is negative.

Sample size	Fig. 2: Between 2 and 5 biological replicates were used to compare the growth rate.<br>Fig. 3a: A culture starting from one biological replicate was plated in different dilutions to enable transformation efficiency calculation. Between 1 and 10 plates per strain were counted.<br>Fig. 3b: Three biological replicates were analysed for their HOC1 mRNA transcript levels.<br>Fig. 5b: 24 biological replicates were analysed for each strain, except for NCYC 2543 where only 11 and 9 clones were grown for pGAP and pAOX1.<br>Extended Data Fig. 2: End ODs were determined from two biological replicates and three technical replicates per strain.
Data exclusions	Fig. 3a: Clones on the dilution plates were only counted when they appeared as individual colonies.<br>Fig. 3b: For NRRL Y-11430 and NCYC 2543 hoc1tr-1 one replicate was removed for both primer pairs due to a technical issue with the prepared cDNA.<br>Fig. 5b: Data from wells were excluded when no expression was observed on SDS-PAGE (Fig. 5a), assuming these clones contain no expression cassette, or due to a technical issue during the ELISA procedure.
Replication	For each experiment, we included enough biological and/or technical replicates to ensure a confident interpretation of the results.
Randomization	NA
Blinding	NA

Behavioural & social sciences study design

All studies must disclose on these points even when the disclosure is negative.

Study description	NA
Research sample	NA
Sampling strategy	NA
Data collection	NA
Timing	NA
Data exclusions	NA
Non-participation	NA
Randomization	NA

Ecological, evolutionary & environmental sciences study design

All studies must disclose on these points even when the disclosure is negative.

Study description	NA
Research sample	NA
Sampling strategy	NA
Data collection	NA
Timing and spatial scale	NA
Data exclusions	NA
Reproducibility	NA
Randomization	NA
Blinding	NA

Did the study involve field work? ☐ Yes ☐ No

Field work, collection and transport

Field conditions	NA
Location	NA
Access & import/export	NA
Disturbance	NA

Reporting for specific materials, systems and methods

We require information from authors about some types of materials, experimental systems and methods used in many studies. Here, indicate whether each material, system or method listed is relevant to your study. If you are not sure if a list item applies to your research, read the appropriate section before selecting a response.

Materials & experimental systems

n/a	Involved in the study	
☐	☒	Antibodies
☒	☐	Eukaryotic cell lines
☒	☐	Palaeontology and archaeology
☒	☐	Animals and other organisms
☒	☐	Clinical data
☒	☐	Dual use research of concern
☒	☐	Plants

Methods

n/a	Involved in the study	
☒	☐	ChIP-seq
☐	☒	Flow cytometry
☒	☐	MRI-based neuroimaging

Antibodies

Antibodies used

penta-His antibody in PBS solution (Qiagen, 34660)
MonoRab™ Rabbit Anti-Camelid VHH Antibody coupled to HRP (GenScript, A01861)
mouse monoclonal anti-V5 (AbD Serotec, MCA2892)
rabbit polyclonal anti-FLAG (Sigma-Aldrich, F7425)
goat anti-mouse AF568 (Life Technologies, A-11031)
goat anti-rabbit AF488 (Life Technologies, A11008)

Validation

- penta-His antibody in PBS solution (Qiagen, 34660) - https://www.qiagen.com/us/products/discovery-and-translational-research/protein-purification/tagged-protein-expression-purification-detection/anti-his-antibodies-bsa-free?catno=34660
- MonoRab™ Rabbit Anti-Camelid VHH Antibody coupled to HRP (GenScript, A01861) - https://www.genscript.com/antibody/A01861- MonoRab_Rabbit_Anti_Camelid_VHH_Antibody_HRP_mAb.html
- mouse monoclonal anti-V5 (AbD Serotec (now BioRad), MCA2892) - https://www.bio-rad-antibodies.com/monoclonal/viral-v5-tag-antibody-sv5-pk2-mca2892.html?f=purified
- rabbit polyclonal anti-FLAG (Sigma-Aldrich, F7425) - https://www.sigmaaldrich.com/BE/en/product/sigma/f7425
- goat anti-mouse AF568 (Life Technologies, A-11031) - https://www.thermofisher.com/antibody/product/Goat-anti-Mouse-IgG-H-L-Highly-Cross-Adsorbed-Secondary-Antibody-Polyclonal/A-11031
- goat anti-rabbit AF488 (Life Technologies, A11008) - https://www.thermofisher.com/antibody/product/Goat-anti-Rabbit-IgG-H-L-Cross-Adsorbed-Secondary-Antibody-Polyclonal/A-11008

Eukaryotic cell lines

Policy information about cell lines and Sex and Gender in Research

Cell line source(s)	NA
Authentication	NA
Mycoplasma contamination	NA
Commonly misidentified lines (See ICLAC register)	NA

Palaeontology and Archaeology

Specimen provenance	NA
Specimen deposition	NA
Dating methods	NA

☐ Tick this box to confirm that the raw and calibrated dates are available in the paper or in Supplementary Information.

Ethics oversight	NA

Note that full information on the approval of the study protocol must also be provided in the manuscript.

Animals and other research organisms

Policy information about studies involving animals; ARRIVE guidelines recommended for reporting animal research, and Sex and Gender in Research

Laboratory animals	NA

Wild animals	NA
Reporting on sex	NA
Field-collected samples	NA
Ethics oversight	NA

Note that full information on the approval of the study protocol must also be provided in the manuscript.

Clinical data

Policy information about clinical studies

All manuscripts should comply with the ICMJE guidelines for publication of clinical research and a completed CONSORT checklist must be included with all submissions.

Clinical trial registration	NA
Study protocol	NA
Data collection	NA
Outcomes	NA

Dual use research of concern

Policy information about dual use research of concern

Hazards

Could the accidental, deliberate or reckless misuse of agents or technologies generated in the work, or the application of information presented in the manuscript, pose a threat to:

No	Yes	
☒	☐	Public health
☒	☐	National security
☒	☐	Crops and/or livestock
☒	☐	Ecosystems
☒	☐	Any other significant area

Experiments of concern

Does the work involve any of these experiments of concern:

No	Yes	
☒	☐	Demonstrate how to render a vaccine ineffective
☒	☐	Confer resistance to therapeutically useful antibiotics or antiviral agents
☒	☐	Enhance the virulence of a pathogen or render a nonpathogen virulent
☒	☐	Increase transmissibility of a pathogen
☒	☐	Alter the host range of a pathogen
☒	☐	Enable evasion of diagnostic/detection modalities
☒	☐	Enable the weaponization of a biological agent or toxin
☒	☐	Any other potentially harmful combination of experiments and agents

Plants

Seed stocks: NA

Novel plant genotypes: NA

Authentication: NA

ChIP-seq

Data deposition

☐ Confirm that both raw and final processed data have been deposited in a public database such as GEO.

☐ Confirm that you have deposited or provided access to graph files (e.g. BED files) for the called peaks.

Data access links
May remain private before publication.: NA

Files in database submission: NA

Genome browser session (e.g. UCSC): NA

Methodology

Replicates: NA

Sequencing depth: NA

Antibodies: NA

Peak calling parameters: NA

Data quality: NA

Software: NA

Flow Cytometry

Plots

Confirm that:

☒ The axis labels state the marker and fluorochrome used (e.g. CD4-FITC).

☒ The axis scales are clearly visible. Include numbers along axes only for bottom left plot of group (a 'group' is an analysis of identical markers).

☒ All plots are contour plots with outliers or pseudocolor plots.

☒ A numerical value for number of cells or percentage (with statistics) is provided.

Methodology

Sample preparation: Clones that were determined to have one integrated copy of the surface display construct were inoculated in BMGY supplemented with 50 μg/ml Zeocin in technical triplicate, and grown for 24 h at 28 °C while shaking at 200 rpm. Afterwards, the cultures were transferred to BMMY supplemented with 50 μg/ml Zeocin, set to an OD600 of 10/ml, and further grown for 24 h at 28 ºC while shaking at 200 rpm. After 12 h, an additional 1% methanol was spiked into the cultures. After induction, the cells were harvested by centrifugation at 1500g for 5 min and washed 3 times with ice-cold washing buffer (PBS+1mM EDTA, pH 7.2+1 Complete Inhibitor EDTA-free tablet (Roche) per 50ml buffer). Cells were kept on ice during the entire staining procedure. Unstained controls, single-stain controls and an empty vector control were taken along.
Cells were stained at an OD600 of2 with mouse monoclonal anti-V5 (1/500, AbD Serotec MCA2892) and rabbit polyclonal anti-FLAG (1/200, Sigma-Aldrich F7425) in ice-cold staining buffer (wash buffer+0.5mg/ml bovine serum albumin) for 1 h at 4 °C. Afterwards, cells were washed 3 times with ice-cold staining buffer and stained with goat anti-mouse AF568 (1/250, Life Technologies A-11031), goat anti-rabbit AF488 (1/500, Life Technologies A11008) and Live/Death stain eFluor506 (1/1000,

	Thermo Fischer Scientific) for 1 h at 4 °C. Cells were washed 3 times with ice-cold staining buffer before analysis on a BD FACSMelodyTM instrument.
Instrument	BD FACSMelody
Software	FlowJo
Cell population abundance	NRRL Y-11430: 99.6%, 99.3%, 98.1% (Clone1 reps1, 2, 3), 99.7%, 99.8%, 99.3% (Clone2 reps 1, 2, 3) of over 10000 events per sort.<br>NCYC 2543: 99.4%, 99.2%, 98.9% (Clone1 reps1, 2, 3), 98.7%, 99.6%, 98.1% (Clone2 reps 1, 2, 3) of over 10000 events per sort.<br>OPENPichia: 99.9%, 99.7%, 100% (Clone1 reps1, 2, 3), 99.9%, 99.4%, 99.9% (Clone2 reps 1, 2, 3) of over 10000 events per sort.
Gating strategy	. First, debris is gated out, then single cells are gated using both the side scatter (SSC) and the forward scatter (FSC), and finally living cells are gated by using the Live/Death stain eFluor506. The resulting living single Pichia cells are analysed based on FLAG and V5 signal. The gates of IV and V were determined based on non-stained and single-stained controls. Further details can be found in Extended Data Figures 5 and 6.

☒ Tick this box to confirm that a figure exemplifying the gating strategy is provided in the Supplementary Information.

Magnetic resonance imaging

Experimental design

Design type	NA
Design specifications	NA
Behavioral performance measures	NA

Acquisition

Imaging type(s)	NA
Field strength	NA
Sequence & imaging parameters	NA
Area of acquisition	NA

Diffusion MRI ☐ Used ☐ Not used

Preprocessing

Preprocessing software	NA
Normalization	NA
Normalization template	NA
Noise and artifact removal	NA
Volume censoring	NA

Statistical modeling & inference

Model type and settings	NA
Effect(s) tested	NA

Specify type of analysis: ☐ Whole brain ☐ ROI-based ☐ Both

Statistic type for inference<br>(See Eklund et al. 2016)	NA
Correction	NA

Models & analysis

n/a	Involved in the study
☒	☐ Functional and/or effective connectivity
☒	☐ Graph analysis
☒	☐ Multivariate modeling or predictive analysis

Functional and/or effective connectivity: NA

Graph analysis: NA

Multivariate modeling and predictive analysis: NA
