# Supplementary material for: OPENPichia: licence-free Komagataella phaffii chassis strains and toolkit for protein expression
Source: Nat Microbiol. 2024 Mar 4;9(3):864–76. doi: 10.1038/s41564-023-01574-w (PMC10914597; doi:10.1038/s41564-023-01574-w)

Claes_Unmodified_Gels_ED_Fig7.pdf

Unmodified SDS-PAGE Extended Data Fig. 7C

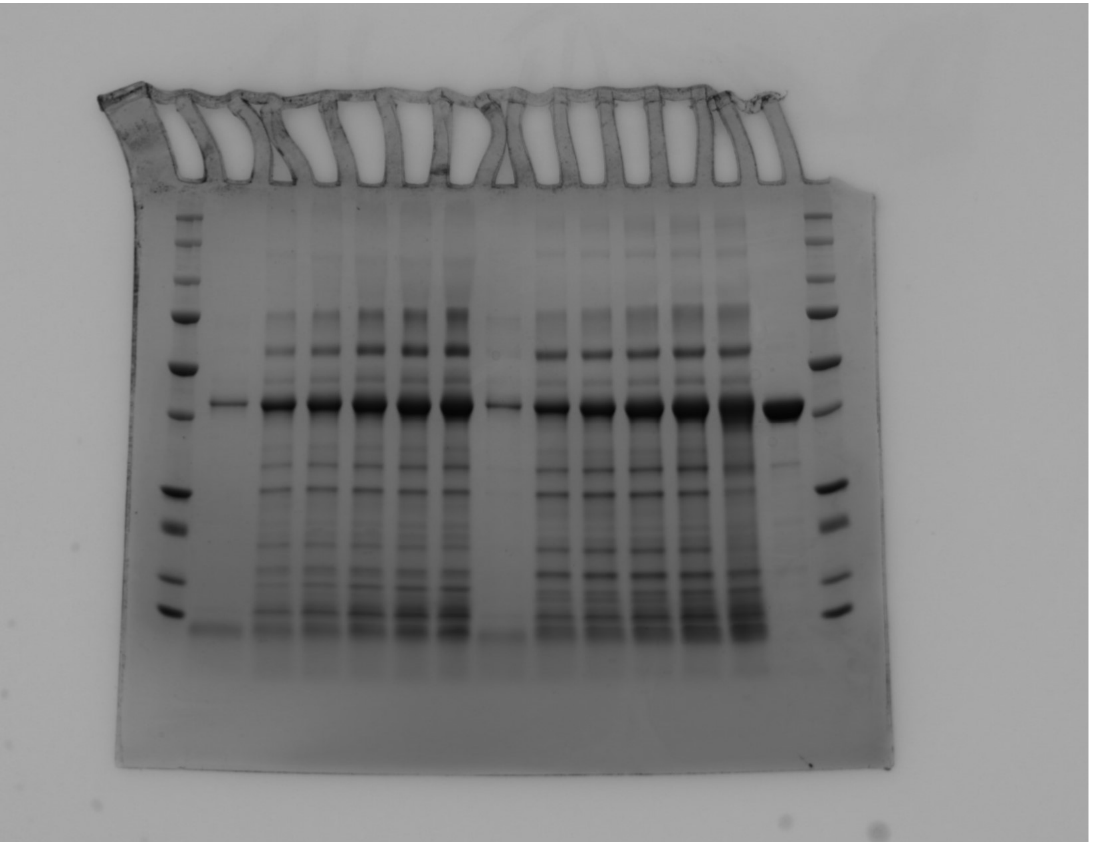

Unmodified SDS-PAGE Extended Data Fig. 7D

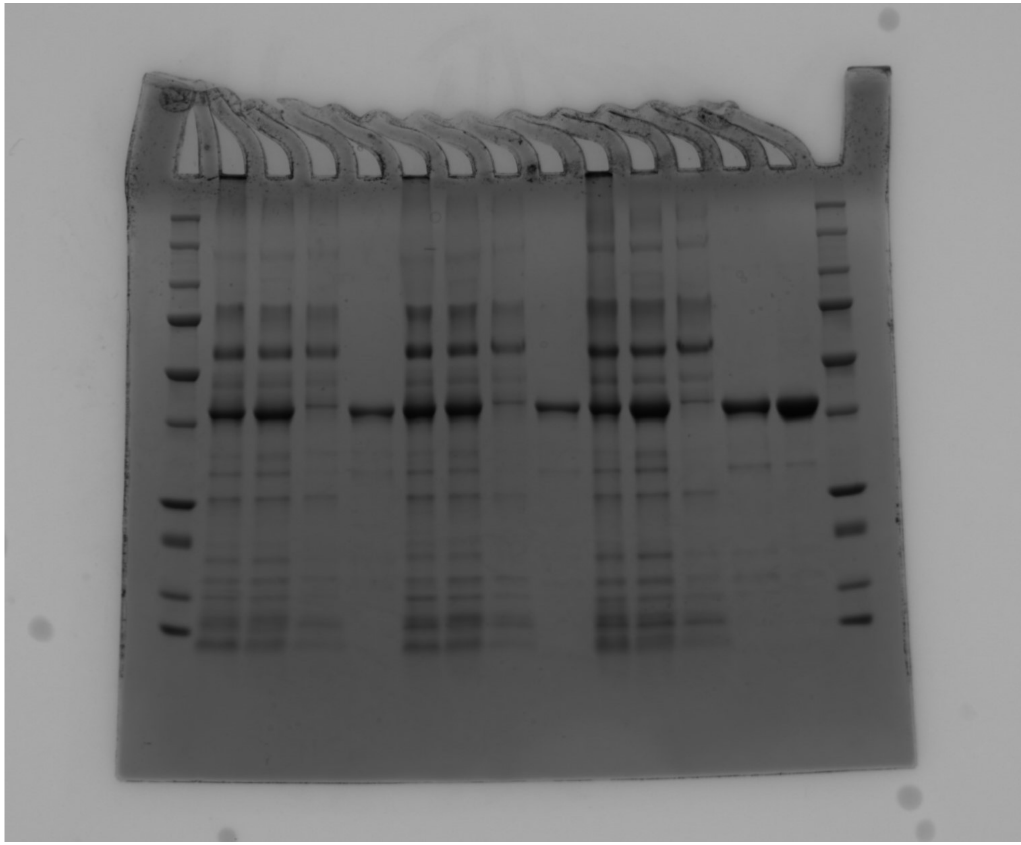

Supplement: Supplementary file 12 — Unprocessed SDS–PAGE gels. [file 41564_2023_1574_MOESM12_ESM.pdf]
